# The role of oxidative stress in modulating mortality risk across the hypertension control cascade

**DOI:** 10.3389/fcvm.2025.1621911

**Published:** 2025-09-08

**Authors:** Weihao Liu, Chunyang Hou, Hongjie Wang, Hao Du, Xianyu Dai, Yu Jiang, Yuchuan Hou

**Affiliations:** Department of Urology, The First Hospital of Jilin University, Changchun, Jilin, China

**Keywords:** oxidative stress, inflammation, hypertension control cascade, cardiovascular mortality, Oxidative Balance Score

## Abstract

**Background:**

The role of oxidative stress in hypertensive populations has not yet been fully elucidated. This study examines the association between the Oxidative Balance Score (OBS) and all-cause and cardiovascular mortality under different hypertension control cascade outcomes while assessing mediation by low-grade systemic inflammation and multi-organ function.

**Methods:**

This cohort study analyzed 1999–2018 NHANES data, with mortality outcomes from the National Death Index (NDI). It encompassed U.S. adults with hypertension. OBS consists of 20 nutrition and lifestyle factors. Low-grade systemic inflammation (NLR, SIRI) and multi-organ function (eGFR, UACR, FIB-4 index, SUA) were examined as potential mediators. Statistical analyses included Kaplan–Meier analysis, Cox models, restricted cubic splines (RCS), subgroup analyses, and mediation analysis.

**Results:**

Participants in the highest OBS quartile (Q4) exhibited lower all-cause mortality (HR: 0.72; 95% CI: 0.59–0.88; *P* = 0.001) and cardiovascular mortality (HR: 0.64; 95% CI: 0.42–0.99; *P* = 0.044) than Q1 after adjusting for confounders. The OBS-mortality association varied by hypertension control status, with greater risk reduction in controlled hypertension (Q4 vs. Q1, HR: 0.43; 95% CI: 0.27–0.69; *P* < 0.001) than in uncontrolled hypertension (Q4 vs. Q1, HR: 0.82; 95% CI: 0.66–0.87; *P* < 0.001). A significant interaction was observed between OBS and hypertension control status (*P* for interaction = 0.017 and 0.026), corroborated by sensitivity analyses (*P* for interaction = 0.025). Sensitivity analyses confirmed Nutrition-OBS reduced all-cause mortality by 31%, and Lifestyle-OBS decreased cardiovascular mortality by 45%. RCS analyses verified the inverse OBS-mortality relationship, with mediation analysis revealing partial mediation through low-grade systemic inflammation and multi-organ function.

**Conclusions:**

A higher OBS is associated with lower all-cause and cardiovascular mortality under different hypertension control cascade outcomes, with a more pronounced effect in controlled hypertension. This relationship is partially mediated through systemic inflammation and multi-organ function.

## 1 Introduction

Hypertension is the main driver of cardiovascular disease and premature death worldwide (1). Effective management of blood pressure has demonstrated a reduction in mortality among individuals with hypertension (2). The management of hypertension emphasizes a comprehensive approach, with evolving strategies continuously refined in accordance with updated clinical guidelines (3–5). Nonetheless, despite continual progress in hypertension management, numerous patients persist in experiencing suboptimal quality of life (6). A major contributing factor is the progression of hypertension, which is frequently accompanied by multi-organ dysfunction. Furthermore, the mechanisms underlying impaired quality of life in hypertensive individuals remain incompletely elucidated (7). Although extensive research has been conducted on the molecular mechanisms of hypertension, there is a scarcity of high-quality, large-scale clinical research investigating key determinants of quality of life in this population.

The prevailing clinical approach to hypertension management is based on the hypertension control cascade strategy, which provides a systematic approach to comprehending and intervening at various stages of hypertension (8). This strategy encompasses three key components: awareness, treatment, and control. First, individuals must recognize their hypertensive status to receive appropriate treatment recommendations. Subsequently, the implementation of effective therapeutic regimens is crucial for attaining optimal blood pressure control (3, 8). The hypertension control cascade has been shown to markedly diminish the likelihood of cardiovascular incidents and enhance patient outcomes (9). However, mortality risk differs among individuals at different stages within the control cascade, necessitating tailored intervention strategies for distinct hypertensive populations (10–12). This variability further underscores the complexity and heterogeneity of the mechanisms driving hypertension progression.

Oxidative stress, along with associated redox signaling pathways, critically influences the pathogenesis and progression of hypertension at molecular, cellular, and systemic scales. These mechanisms drive endothelial damage, vascular dysfunction, remodeling of cardiovascular tissues, renal injury, hyperactivation of the sympathetic nervous system, immune cell stimulation, and chronic systemic inflammatory responses (13–15). Among them, the renin-angiotensin-aldosterone system (RAAS) serves as an indispensable regulator of the pathophysiological processes underlying hypertension, primarily through the actions of angiotensin II (Ang II) (16). By stimulating NADPH oxidases (NOXs), Ang II facilitates an excessive generation of reactive oxygen species (ROS), enhancing oxidative stress and vascular dysfunction (17). Excessive ROS not only directly damages vascular endothelium, but also triggers inflammatory responses, further exacerbating vascular dysfunction. Additionally, ROS inhibits the synthesis and bioavailability of nitric oxide (NO), resulting in compromised vasodilation, heightened peripheral vascular resistance, and subsequent blood pressure elevation (18). Moreover, ROS promotes vascular smooth muscle cell proliferation, inflammation, and fibrosis, resulting in arterial wall thickening, reduced elasticity, and decreased vascular compliance, all of which expedite hypertension progression (19, 20). Furthermore, research has revealed a robust association between oxidative stress levels and the extent of target organ damage in individuals with hypertension (21–24). This underscores the crucial involvement of oxidative stress in hypertension pathogenesis and emphasizes targeting oxidative stress as a potential therapeutic strategy for mitigating hypertension and its associated organ damage.

Multiple factors influence oxidative stress levels within the body. Pro-oxidant factors generally encompass behaviors such as tobacco use, ethanol intake, excessive adiposity, and the consumption of specific pro-oxidant nutrients, whereas antioxidant factors encompass distinct antioxidant nutrients and engagement in physical activity (25). Nonetheless, the impact of inherent individual variables on oxidative/antioxidant homeostasis remains constrained (25). The Oxidative Balance Score (OBS) was established as a comprehensive index to assess the interplay of pro-oxidants and antioxidants influenced by nutritional intake and way of living, hence assessing an individual's oxidative/antioxidant state (25). OBS is determined by 20 nutritional and lifestyle components classified as either pro-oxidant or antioxidant (25). In general, an elevated OBS indicates a greater predominance of antioxidants over pro-oxidant factors, whereas a lower OBS signifies a relative imbalance characterized by heightened pro-oxidant activity. Consistent with mechanistic research, OBS has been demonstrated to be linked to multiple hypertension-related conditions, including ischemic heart disease, stroke, chronic kidney disease, other cardiovascular diseases (CVD), and cognitive impairment (26–30). However, whether OBS plays a critical role in target organ involvement during hypertension progression remains incompletely elucidated.

The management of hypertension as a chronic condition may be significantly influenced by an individual's lifestyle and dietary practices beyond the clinical setting environment (31). Therefore, a systematic assessment of the influence of the Oxidative Balance Score (OBS) on hypertensive patients holds considerable clinical and research value for elucidating the multifactorial mechanisms underlying hypertension progression. The present investigation represents the first attempt to incorporate the hypertension control cascade framework to illuminate the relationship between oxidative balance score (OBS) and both all-cause and cardiovascular mortality outcomes. This integration provides novel insights into mortality risk stratification among hypertensive populations. Furthermore, we examine whether low-grade systemic inflammation and multi-organ function mediate the relationship between OBS and all-cause mortality risk. Our findings enhance the understanding of how oxidative stress levels influence survival across varying hypertension control statuses, providing a theoretical basis for identifying high-risk populations requiring intensified monitoring and intervention. Enhancing a comprehensive understanding of this intricate chronic disease, this investigation offers essential insights into the multidimensional mechanisms of hypertension.

## 2 Materials and methods

### 2.1 Study population

**Figure 1:**
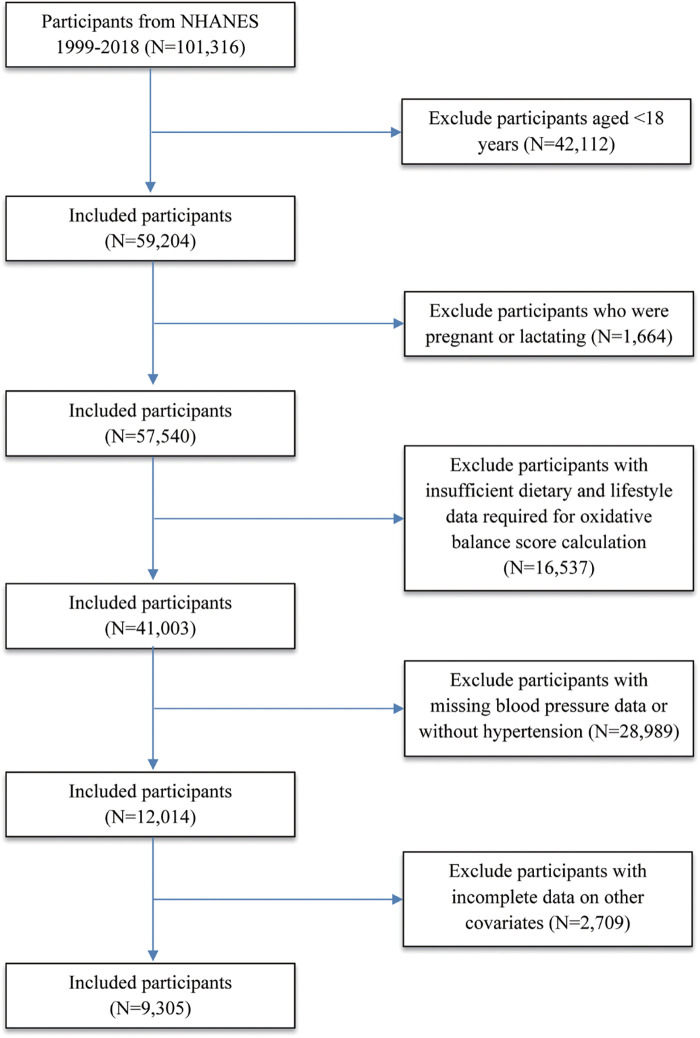
Flowchart illustrating the enrollment and selection of NHANES 1999–2018 participants included in the final analysis (*N* = 9,305).

This cohort study analyzed ten consecutive cycles (1999–2018) of NHANES, a cross-sectional survey employing a stratified, multistage probability sampling approach to generate nationally representative data on the U.S. civilian, non-institutionalized population (32). The NHANES study protocol received approval from the NCHS Ethics Review Board, and all participants provided written informed consent. All procedures were conducted in accordance with the guidelines of the Declaration of Helsinki. As this study utilized publicly available, de-identified data, the Institutional Review Board of Jilin University determined that it was not subject to ethical review and consent obligations (33). The study followed STROBE guidelines for observational research. A total of 101,316 individuals were initially recruited. The subsequent exclusions were implemented: (1) Participants younger than 18 years (*n* = 42,112) (2) Pregnant or lactating individuals (*n* = 1,664); (3) Participants with missing blood pressure data (*n* = 8,515) (4) Individuals with insufficient dietary and lifestyle data required for OBS calculation (*n* = 16,537) (5) Non-hypertensive participants (*n* = 20,474) (6) Participants with missing data on key covariates (*n* = 2,709). After these exclusions, there were ultimately 9,305 participants included in the final analytical cohort (Figure 1).

### 2.2 Exposure variable

Our construction of the Oxidative Balance Score (OBS) followed previously established methodologies in the field (25, 34, 35). The Oxidative Balance Score (OBS) consists of 20 elements, with 16 classified as nutrients and 4 as lifestyle variables, encompassing 5 pro-oxidant and 15 antioxidant components (34, 35). Dietary information was obtained through the initial 24-hour dietary recall interview, encompassing the intake of key nutrients such as dietary fiber, carotenoids (expressed as retinol equivalents), antioxidant vitamins (*α*-tocopherol equivalents), and minerals like calcium and magnesium, among others (Supplementary Table 1). The evaluation encompassed various lifestyle-associated determinants, including physical activity levels, body mass index (BMI), alcohol consumption, and smoking habits, with smoking exposure quantified through serum cotinine concentrations. Within this framework, BMI, total fat intake, iron levels, alcohol intake, and smoking were categorized as pro-oxidative elements, while all remaining factors were designated as contributors to antioxidant capacity. Consistent with methods described previously for calculating OBS (34, 35), alcohol intake was categorized into three distinct levels: non-drinkers (score = 2), light-to-moderate drinkers (women: 0–15 g/day; men: 0–30 g/day, score = 1), and heavy drinkers (women: ≥15 g/day; men: ≥30 g/day, score = 0). The remaining components were assigned scores according to tertiles stratified by sex. Scores of antioxidant components were allocated as 0, 1, and 2, corresponding respectively to the lowest, intermediate, and highest sex-specific tertiles. For pro-oxidant components, the scoring system was applied inversely. This unweighted approach, based on population-specific tertiles, is a common and validated practice in OBS research, as a systematic review by Hernández-Ruiz et al. (2019) concluded that it yields results comparable to more complex weighted models (35). The whole OBS was computed by aggregating the individual values of all components, with elevated scores signifying increased antioxidant exposure (Supplementary Table 1). Patients were stratified into four quartiles according to the distribution of OBS within the hypertensive population.

### 2.3 Hypertension definition and the definition of hypertension control cascade

Systolic blood pressure (SBP) and diastolic blood pressure (DBP) were derived as the average of up Unlike previous studiesto three sequential blood pressure readings. Hypertension was defined as SBP ≥130 mmHg or DBP ≥80 mmHg, or self-reported ongoing antihypertensive medication usage, irrespective of measured blood pressure levels. Uncontrolled hypertension was additionally delineated depending on the 2017 ACC/AHA guidelines, irrespective of antihypertensive medication use (36). Hypertension awareness was assessed by asking participants: “Has a doctor or other health professional ever told you that you have hypertension, also called high blood pressure?” Individuals who responded “yes” were classified as being aware of their hypertensive status. Treatment recommendations were established following the 2017 ACC/AHA guidelines. Individuals diagnosed with hypertension were considered eligible for lifestyle modification and antihypertensive therapy if they fulfilled any of the subsequent three requirements: (1) current use of antihypertensive medication; (2) stage 2 hypertension (SBP ≥140 mmHg or DBP ≥90 mmHg); or (3) stage 1 hypertension (SBP 130–139 mmHg or DBP 80–89 mmHg) accompanied by predisposing conditions, including a prior diagnosis of CVD, an atherosclerotic cardiovascular disease (ASCVD) risk score of ≥10%, or age of ≥65 years. Participants identified as needing only lifestyle modification were those with stage 1 hypertension and a reduced CVD risk (ASCVD score <10%). Individuals lacking awareness of their hypertensive status were deemed unsuitable for any treatment recommendations (37). The hypertension control cascade was evaluated through a stepwise method, calculating the sequential phases (38) (Table 1).

**Table 1:** Baseline characteristics of U.S. adults with hypertension (*N* = 9,305), NHANES 1999–2018.

Characteristic	Weighted mean (SE) or weighted percentage (unweighted *n*)^a^
Age, years	53.1 (0.3)
Age group, years	
<45	29.9 (2,491)
45–64	44.1 (3,685)
≥65	26.0 (3,129)
Female sex	43.1 (3,921)
Race/ethnicity	
Mexican American	5.1 (1,142)
Other Hispanic	3.6 (551)
Non-Hispanic White	73.7 (4,677)
Non-Hispanic Black	10.9 (2,116)
Other Race	6.6 (819)
Family-poverty-income ratio	
≤1.30	13.6 (1,897)
>1.30 and ≤3.50)	31.4 (3,203)
>3.50	49.0 (3,525)
Missing data	6.0 (680)
Educational attainment	
Less than high school	11.2 (1,716)
High school graduate, general educational development, or equivalent	22.5 (2,140)
Some college or associates degree	32.7 (2,863)
College graduate or above	33.3 (2,551)
Missing data	0.2 (35)
Health insurance	
Insured	88.8 (8,054)
Uninsured	10.9 (1,221)
Missing data	0.3 (30)
Health care visits in the past year, No.	
0	11.5 (1,099)
1	16.9 (1,519)
≥2	71.5 (6,676)
Marital status	
Married	60.5 (5,343)
Single	33.2 (3,372)
Living with a partner	5.1 (480)
Missing data	1.2 (110)
Smoking	
Yes	47.0 (4,322)
No	53.0 (4,983)
Drinking	
Yes	73.0 (6,379)
No	21.8 (2,365)
Missing data	5.2 (561)
Diabetes	
Yes	14.5 (1,781)
No	85.5 (7,524)
Hypertension	
Controlled hypertension	17.7 (1,665)
Unaware, not recommended treatment	46.7 (4,027)
Aware, met criteria for lifestyle modifications	8.7 (651)
Aware, met criteria for lifestyle modifications and medication	6.8 (653)
Aware, met criteria for lifestyle modifications and medication, and is currently taking BP medication	20.1 (2,309)
History of cardiovascular disease	8.9 (992)
History of stroke	2.8 (337)

^a^ Data are presented as weighted mean (SE) for continuous variables and weighted percentage (unweighted N) for categorical variables.

### 2.4 Mediation variables

This study integrated essential biomarkers of systemic inflammation, including the neutrophil-to-lymphocyte ratio (NLR) and the systemic inflammatory response index (SIRI). The calculations for NLR and SIRI were derived from peripheral blood cell counts, with SIRI calculated as (neutrophils × monocytes)/lymphocytes (39). Estimated glomerular filtration rate (eGFR) and urinary albumin-to-creatinine ratio (UACR) were used to assess renal function (Supplementary Information). Glomerular filtration rate (GFR) is a primary indicator of kidney function, reflecting the efficiency of renal clearance of waste products from the blood. The estimated GFR (eGFR), commonly used in clinical assessments, was calculated based on serum creatinine levels (40). UACR, recognized for its elevated specificity and sensitivity, is essential for the early identification of chronic kidney disease (CKD) (41). To assess hepatic damage and fibrosis, we utilized the Fibrosis-4 (FIB-4) index, a serum biochemical marker with a specificity and sensitivity threshold greater than 0.8 for liver fibrosis detection (42) (Supplementary Information). FIB-4 is widely used in disease monitoring for fibrosis staging and liver function assessment. Serum uric acid (SUA) is associated with multiple organ pathologies and metabolic functions. SUA was assessed using a timed endpoint method. A detailed description of laboratory methods for measuring blood cell counts, serum creatinine, urinary albumin, urinary creatinine, and other biochemical markers can be accessed through the official websites of the CDC and NHANES.

### 2.5 Outcomes and covariates

The primary outcomes of this study were all-cause mortality and cardiovascular mortality. Mortality data were obtained by linking the cohort database to the National Death Index (NDI), with follow-up continuing until December 31, 2019. The time-to-event was determined from the date of the NHANES examination to either the recorded date of death or the end of follow-up (December 31, 2019), whichever came first. Cardiovascular mortality was identified according to codes I00–I09, I11, I13, and I20–I51 from the International Classification of Diseases, 10th Revision (ICD-10). Building on prior literature, we integrated a range of covariates into our analysis of the NHANES study (29). In this analysis, covariates encompassed age, self-reported race/ethnicity, and sex. Educational attainment was classified as less than high school, high school graduate or equivalent, some college or associate degree, and college graduate or higher. Marital status was categorized as married, single, or living with a partner. The poverty-to-income ratio was categorized as ≤1.30, 1.3–3.50, and >3.50. Health insurance status (insured vs. uninsured) and healthcare utilization (0, 1, or ≥2 visits) were also included. Smoking history was defined as a lifetime consumption of at least 100 cigarettes. Drinking history was characterized as the intake of a minimum of 12 alcoholic beverages annually. Participants were classified as having diabetes mellitus based on self-reported diagnosis, hemoglobin A1c (HbA1c) levels ≥6.5%, or fasting plasma glucose levels ≥126 mg/dl. A history of clinical cardiovascular disease (CVD) was determined based on self-reported diagnoses of coronary heart disease, congestive heart failure, acute myocardial infarction, angina, or stroke.

### 2.6 Statistical analysis

Considering the intricate sampling design of NHANES, all analyses in this study accounted for sampling weights, clustering, and stratification to enhance representativeness and minimize bias. This study utilized the WTDRD1 (Dietary Day Weight) from the NHANES dataset to calculate the 10-year combined dietary weight (weight10). Specifically, for the 1999–2000 and 2001–2002 periods, the calculation formula was weight10 = WTDRD1 × (2/10), whereas for the 2003–2018 period, it was weight10 = WTDRD1 × (1/10). Baseline data were presented as weighted means (SE) for continuous variables and weighted percentages (unweighted N) for categorical variables. For covariates with missing data (<20% of total data), missing values were imputed using the random forest (missForest) method (43). Temporal analyses and Kaplan–Meier survival curves were utilized to assess variations in survival outcomes. Three multivariable-adjusted Cox proportional hazards models (Models 1–3) were constructed to investigate the relationships of OBS with all-cause and cardiovascular mortality among participants with controlled and uncontrolled hypertension. Model 1 adjusted for age (by incorporating the variable into the statistical model). Model 2 was adjusted for demographic factors, building upon age adjustments by further accounting for sex and race/ethnicity. Socioeconomic indicators (educational attainment, poverty-to-income ratio, marital status, and insurance status) and lifestyle-related factors (smoking behavior, alcohol consumption, healthcare utilization, and history of diabetes) were additionally adjusted in Model 3. To visually evaluate potential nonlinear relationships between OBS and mortality, restricted cubic spline (RCS) curves with three knots were generated, with adjustments for covariates (based on Model 3). Subgroup analyses and interaction tests were conducted to explore the association between OBS and outcomes across different subgroups, building upon the covariates adjusted in Model 3. To account for the issue of multiple comparisons in our exploratory interaction analyses, we applied the Benjamini-Hochberg procedure to control the False Discovery Rate (FDR). The interaction tests were grouped into four logical families based on the specific scientific question each analysis intended to address, and FDR correction was performed separately for each family. All subgroup analyses used the lowest quartile (Q1) as the reference and adjusted for all covariates except for the stratifying variable, to evaluate whether these stratifying variables influenced the robustness of the results. Regression analyses were performed separately for controlled and uncontrolled hypertension subgroups based on blood pressure control status. Sensitivity analyses were conducted to assess the robustness of the primary findings. OBS was stratified into Nutrition-OBS and Lifestyle-OBS for separate Cox regression analyses. To further ensure robustness, participants with controlled hypertension (SBP ≤130 mmHg and DBP ≤80 mmHg) were excluded from the primary analysis, and the uncontrolled hypertension population was further stratified into four subgroups based on hypertension control cascade outcomes. We examined interactions between hypertension control cascade outcomes and overall OBS, Nutrition-OBS, and Lifestyle-OBS, respectively. A sensitivity analysis was conducted using multivariable Cox regression for OBS and its relationship with two mortality types (all-cause and cardiovascular mortality) after excluding samples with missing covariates (*n* = 8,039) to assess the consistency of results before and after imputation. Mediation analysis was conducted using the R package “mediation”, with full covariate adjustment for the constructed models. The results included indirect effects, total effects, proportion mediated, and p-values.

All statistical analyses were conducted using R version 4.4.2, with two-sided *p*-values <0.05 indicating statistical significance.

## 3 Results

### 3.1 Baseline characteristics

This research finally encompassed 9,305 hypertensive patients. Among the study population, 43.1% were female, and had an average age of 53.1 years (SE 0.3), with 73.7% being non-Hispanic White (Table 1). Approximately 49% of participants had a poverty-to-income ratio greater than 3.5, while 11.2% had an education level below high school. Lack of health insurance was reported by 10.9% of the study population, and 71.5% had attended at least two healthcare visits. The majority of participants (73.0%) had a history of alcohol consumption, while 53% reported never smoking. Within the study population, 14.5% had both hypertension and diabetes, and 8.9% had a history of CVD. A history of stroke was reported in only 2.8% of participants. A substantial proportion (82.3%) of participants had uncontrolled hypertension. Among those with uncontrolled hypertension, 46.7% of the total study population were unaware of their hypertensive status. Notably, 35.6% of participants were aware of their hypertension but remained in the uncontrolled hypertension category, regardless of whether they had attempted lifestyle modifications or taken antihypertensive medication. Furthermore, 20.1% of individuals with uncontrolled hypertension had implemented both lifestyle changes and pharmacological treatment.

### 3.2 Associations of the overall OBS, nutrition-OBS, and lifestyle-OBS with all-cause and cardiovascular mortality

**Figure 2:**
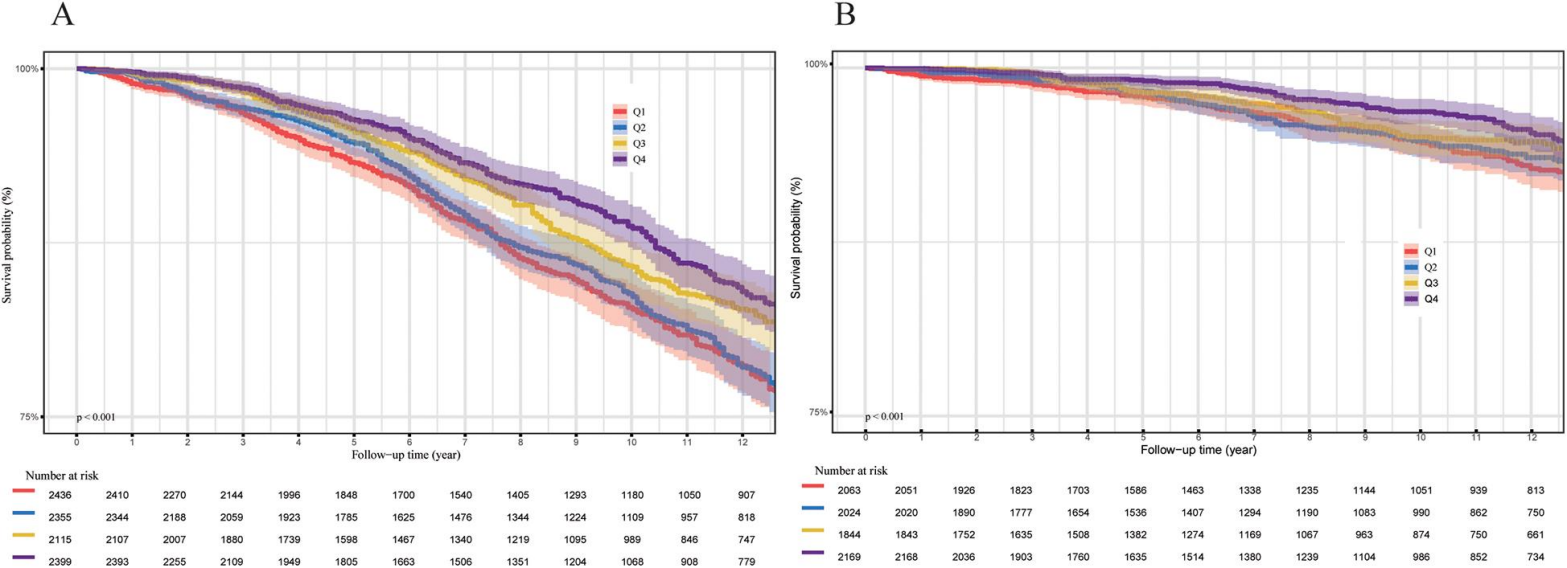
**(A)** All-cause mortality (*N* = 9,305). **(B)** Cardiovascular mortality (*N* = 8,100). *P*-values were calculated using the log-rank test.

**Figure 3:**
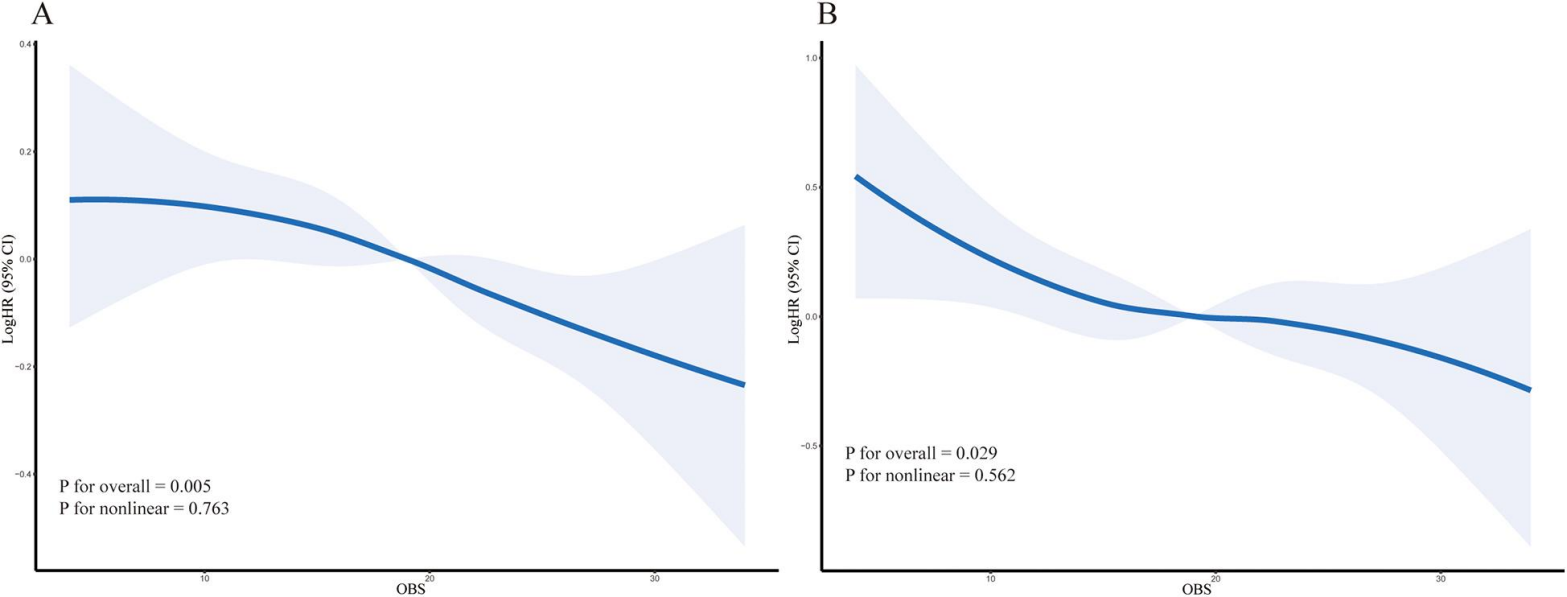
The solid line represents the multivariable-adjusted hazard ratio (HR) and the shaded area represents the 95% confidence interval (CI), estimated by restricted cubic splines. **(A)** All-cause mortality. **(B)** Cardiovascular mortality. P for overall association and P for non-linearity are shown on the plots.

During a median follow-up period of 122 months, a total of 1,620 participants (13.3%) died, including 415 (3.3%) from cardiovascular disease. The hypertensive population was stratified into four quartiles (Q1, Q2, Q3, and Q4) based on OBS levels. The Kaplan–Meier survival analysis revealed a statistically significant disparity in all-cause and cardiovascular mortality across these quartiles *(P* < 0.001, Figure 2). Individuals in the highest OBS quartile (Q4) experienced the greatest survival advantage, while those in the lowest quartile (Q1) faced the highest mortality risk (Q4 vs. Q1, *P* < 0.001). In the fully adjusted multivariate analysis (Model 3), which accounted for additional covariates, each one-unit increase in OBS was associated with a 9% decreased risk of all-cause mortality (HR 0.91, 95% CI 0.86–0.96, *P* < 0.001). Compared to Q1, Q4 participants showed a significantly lower all-cause mortality risk (HR 0.72, 95% CI 0.59–0.88, *P* for trend = 0.001). For the completely adjusted Cox regression (Model 3), a 13% reduction in cardiovascular mortality risk was associated with each one-unit increment in OBS (HR 0.87, 95% CI 0.76–0.99, *P* = 0.033). In comparison to Q1, Q4 individuals exhibited a 36% reduced risk of cardiovascular death (HR 0.64, 95% CI 0.42–0.99, P for trend = 0.005) (Table 2). All-cause mortality risk was reduced by 31% in the highest Nutrition-OBS quartile (Q4; HR 0.69, 95% CI 0.56–0.86, *P* < 0.001; Supplementary Table 2), while cardiovascular mortality risk was lower in Q3 (HR 0.69, 95% CI 0.50–0.95, *P* = 0.025). However, those at Q4 level of Lifestyle-OBS showed only a reduction in cardiovascular mortality risk by 45% (HR 0.55, 95% CI 0.34–0.89, *P* = 0.014) (Supplementary Table 3). Restricted cubic spline (RCS) modeling with adjustments for covariates (based on Model 3) demonstrated a negative correlation between OBS and the likelihood of two mortality types (all-cause and cardiovascular mortality) among hypertensive patients (Figure 3).

**Table 2:** Multivariable Cox regression of oxidative balance score and all-cause and cardiovascular mortality in U.S. adults with hypertension (*N* = 9,305 and 8,100).

Characteristic	Model 1				Model 2				Model 3			
	HR^a^	95% CI^a^	*p*-value	p for trend	HR^a^	95% CI^a^	*p*-value	p for trend	HR^a^	95% CI^a^	*p*-value	p for trend
All-cause death				<0.001				<0.001				<0.001
OBS	0.84	0.80, 0.89	<0.001		0.84	0.80, 0.89	<0.001		0.91	0.86, 0.96	<0.001	
OBS (Quartile)												
Q1	Reference	Reference			Reference	Reference			Reference	Reference		
Q2	0.96	0.79, 1.17	0.7		0.95	0.78, 1.15	0.6		1.08	0.90, 1.29	0.4	
Q3	0.81	0.69, 0.94	0.006		0.80	0.69, 0.93	0.004		0.94	0.81, 1.10	0.4	
Q4	0.57	0.48, 0.69	<0.001		0.57	0.48, 0.69	<0.001		0.72	0.59, 0.88	0.001	
Cardiovascular death				<0.001				<0.001				=0.005
OBS	0.79	0.70, 0.88	<0.001		0.78	0.69, 0.88	<0.001		0.87	0.76, 0.99	0.033	
OBS (Quartile)												
Q1	Reference	Reference			Reference	Reference			Reference	Reference		
Q2	0.97	0.70, 1.33	0.8		0.91	0.66, 1.26	0.6		1.10	0.79, 1.52	0.6	
Q3	0.66	0.48, 0.91	0.011		0.63	0.46, 0.88	0.006		0.83	0.59, 1.16	0.3	
Q4	0.48	0.33, 0.70	<0.001		0.48	0.32, 0.70	<0.001		0.64	0.42, 0.99	0.044	

a Indicates that the Hazard Ratios (HR) and 95% Confidence Intervals (CI) were adjusted for the covariates listed. Model 1 adjusted for age. Model 2 adjusted for sex, age, and race/ethnicity. Model 3 adjusted for sex, age, race/ethnicity, educational level, marital status, poverty-to-income ratio, smoking history, alcohol consumption, health insurance status, healthcare utilization, and history of diabetes.

### 3.3 Subgroup and sensitivity analyses

We performed a subgroup analysis to examine the stability and consistency of the identified relationships between OBS and mortality in hypertensive patients (Supplementary Table 4). The findings indicated that the correlation between OBS and mortality was generally aligned with prior observations across the majority of subgroups, demonstrating a negative correlation that was statistically significant. Although some unadjusted *p*-values for interaction were low, none of these interactions remained statistically significant after applying the Benjam-Hochberg FDR correction for multiple comparisons, suggesting no major effect modification. When performing stratified regression analyses based on blood pressure control status, a significant interaction between OBS quartiles and hypertension subgroups was observed (*P* for interaction = 0.017 and 0.026, Table 3). This finding remained robust after FDR correction (q-values = 0.026 and 0.026, respectively). Compared to Q1, a higher OBS was associated with a reduced risk of all-cause and cardiovascular mortality in the controlled hypertension group (HR 0.43, 95% CI 0.27–0.69, *P* < 0.001; HR 0.29, 95% CI 0.11–0.8, *P* < 0.001). In the uncontrolled hypertension subgroup, a similar trend was observed for both all-cause and cardiovascular mortality (HR 0.82, 95% CI 0.66–0.87, *P* < 0.001; HR 0.8, 95% CI 0.5–1.29, *P* < 0.001). The interaction between Nutrition-OBS and blood pressure control status was only marginally significant for cardiovascular mortality risk (*P* = 0.052; Supplementary Tables 5, 6). In sensitivity analyses, we excluded participants with controlled hypertension, restricting the analysis to individuals with uncontrolled hypertension (*n* = 7,640, Table 4 and Supplementary Tables 7, 8). These participants were further stratified into four hypertension control cascade subgroups. The results demonstrated a significant interaction between hypertension control cascade subgroups and OBS quartiles (*P* for interaction = 0.025, FDR-adjusted q-value = 0.05) but did not reach statistical significance for cardiovascular mortality risk. In the subgroup of participants who were unaware of their hypertension and did not meet any treatment recommendations, Q4 exhibited a 10% lower risk of all-cause mortality compared to Q1 (HR 0.90, 95% CI 0.65–1.24, *P* < 0.001). In the subgroup of participants aware of their hypertension and meeting lifestyle modification criteria, Q4 compared to Q1 did not show a statistically significant reduction in all-cause mortality risk. Among those who recognized their hypertension and fulfilled both lifestyle modification and pharmacological treatment criteria, Q3 and Q4 exhibited a 44% and 40% reduction in all-cause mortality risk compared to Q1, respectively (HR 0.56, 95% CI 0.29–1.06, *P* < 0.001; HR 0.60, 95% CI 0.31–1.19, *P* < 0.001). In participants meeting both lifestyle modification and pharmacological treatment criteria and currently taking antihypertensive medication, compared to Q1, the all-cause mortality risks for Q2, Q3, and Q4 were 0.77, 0.79, and 0.54, respectively (HR 0.77, 95% CI 0.59–1.01, *P* < 0.001; HR 0.79, 95% CI 0.59–1.05, *P* < 0.001; HR 0.54, 95% CI 0.39–0.76, *P* < 0.001). In the sensitivity analysis excluding samples with missing covariates, the multivariable Cox regression results for OBS and outcomes were consistent with those obtained using the random forest imputation method (Supplementary Table 9). We further explored the interaction between Nutrition-OBS and Lifestyle-OBS on mortality. No significant interaction was detected for either all-cause mortality (P for interaction = 0.988) or cardiovascular mortality (P for interaction = 0.584), suggesting their effects are additive (Supplementary Table 10).

**Table 3:** Associations of oxidative balance score (OBS) quartile groups with hypertension subgroups stratified by blood pressure control status (all-cause death: *N* = 9,305; cardiovascular death: *N* = 8,100).

Variable	Count	Percent	Levels	Point estimate	Lower	Upper	*P* value	P for interaction	FDR-adjusted q-value
All-cause death									
Hypertension Subgroups	9,305							0.017	0.026
Controlled hypertension	1,665	17.9	Q1	Reference					
			Q2	0.71	0.47	1.07	<0.001		
			Q3	0.79	0.53	1.18	<0.001		
			Q4	0.43	0.27	0.69	<0.001		
Uncontrolled hypertension	7,640	82.1	Q1	Reference					
			Q2	1.19	0.97	1.45	1.695		
			Q3	0.99	0.83	1.18	<0.001		
			Q4	0.82	0.66	0.87	<0.001		
Cardiovascular death									
Hypertension Subgroups	8,100							0.026	0.026
Controlled hypertension	1,442	17.8	Q1	Reference					
			Q2	1.05	0.52	2.12	0.13		
			Q3	0.83	0.37	1.82	<0.001		
			Q4	0.29	0.11	0.8	<0.001		
Uncontrolled hypertension	6,658	82.2	Q1	Reference					
			Q2	1.15	0.77	1.74	0.682		
			Q3	0.84	0.57	1.24	<0.001		
			Q4	0.8	0.5	1.29	<0.001		

Adjusted for sex, age, race/ethnicity, educational level, marital status, poverty-to-income ratio, smoking history, alcohol consumption, health insurance status, healthcare utilization, and history of diabetes.

**Table 4:** Sensitivity analysis excluding participants with controlled hypertension and stratifying into four hypertension control cascade subgroups (all-cause death: *N* = 7,640; cardiovascular death: *N* = 6,658).

Variable	Count	Percent	Levels	Point estimate	Lower	Upper	*P* value	P for interaction	FDR-adjusted q-value
All-cause death									
Hypertension control cascade subgroups	7,640							0.025	0.05
Unaware, not recommended treatment	4,027	52.7	Q1	Reference					
			Q2	1.51	1.09	2.07	2.502		
			Q3	1.12	0.81	1.55	0.698		
			Q4	0.9	0.65	1.24	<0.001		
Aware, met criteria for lifestyle modifications	651	8.5	Q1	Reference					
			Q2	3.86	0.83	17.98	1.72		
			Q3	5.99	1.53	23.51	2.567		
			Q4	1.71	0.36	8.16	0.678		
Aware, met criteria for lifestyle modifications and medication	653	8.5	Q1	Reference					
			Q2	1.18	0.64	2.17	0.535		
			Q3	0.56	0.29	1.06	<0.001		
			Q4	0.6	0.31	1.19	<0.001		
Aware, met criteria for lifestyle modifications and medication, and is currently taking BP medication	2,309	30.2	Q1	Reference					
			Q2	0.77	0.59	1.01	<0.001		
			Q3	0.79	0.59	1.05	<0.001		
			Q4	0.54	0.39	0.76	<0.001		
Cardiovascular death									
Hypertension control cascade subgroups	6,658							0.176	0.176
Unaware, not recommended treatment	3,621	54.4	Q1	Reference					
			Q2	1.49	0.72	3.1	1.074		
			Q3	1.32	0.76	2.3	0.98		
			Q4	0.92	0.56	1.5	<0.001		
Aware, met criteria for lifestyle modifications	631	9.5	Q1	Reference					
			Q2	–	–	–	–		
			Q3	–	–	–	–		
			Q4	–	–	–	–		
Aware, met criteria for lifestyle modifications and medication	562	8.4	Q1	Reference					
			Q2	1.39	0.36	5.41	0.474		
			Q3	0.26	0.06	1.09	<0.001		
			Q4	0.48	0.14	1.62	<0.001		
Aware, met criteria for lifestyle modifications and medication, and is currently taking BP medication	1,844	27.7	Q1	Reference					
			Q2	0.78	0.5	1.22	<0.001		
			Q3	0.58	0.36	0.92	<0.001		
			Q4	0.52	0.27	0.99	<0.001		

Adjusted for sex, age, race/ethnicity, educational level, marital status, poverty-to-income ratio, smoking history, alcohol consumption, health insurance status, healthcare utilization, and history of diabetes.

### 3.4 Mediation analyses

The estimated glomerular filtration rate (eGFR) served as a significant mediator, with a mediation proportion of 2.08% (*P* < 0.001) (Supplementary Table 10 and Figure 1). Urinary albumin-to-creatinine ratio (UACR) also demonstrated a mediation proportion of 1.55% (*P* < 0.001) in this association. Additionally, NLR and SIRI were identified as mediators, with mediation proportions of 2.02% (*P* = 0.040) and 3.17% (*P* < 0.001), respectively. Notably, serum uric acid (SUA) exhibited the highest mediation proportion, accounting for 5.47% of the correlation between OBS and all-cause mortality (*P* = 0.040). Conversely, the mediation effect of the FIB-4 index in this association was not statistically significant.

## 4 Discussion

Oxidative stress is pivotal in the initiation, maintenance, and progression of hypertension, contributing to multiple pathophysiological processes and providing a potential therapeutic target for hypertension management (15, 21). The OBS functions as a proxy measure for systemic oxidative stress levels, facilitating the investigation of the intricate correlation between oxidative stress and mortality risk (25). Unlike previous studies, we found that Nutrition-OBS was associated with reduced all-cause mortality, whereas Lifestyle-OBS primarily mitigated the risk of adverse events related to cardiovascular disease. This divergence may reflect the broader systemic benefits of optimal nutritional status on overall individual health, while lifestyle factors such as physical activity and smoking cessation may exert more specific influences on cardiovascular outcomes. The observation that the Nutrition-OBS association with reduced cardiovascular mortality risk reached statistical significance in Q3 but not the highest quartile (Q4) likely reflects limited statistical power due to fewer cardiovascular events recorded in Q4. The maintenance of hypertension can also be identified and intervened upon through the hypertension control cascade strategy (9). However, the progression of hypertension, driven by persistent oxidative stress, leads to target organ damage and functional impairment through complex and heterogeneous mechanisms (13, 14). Further investigations are required to elucidate these mechanisms while identifying reliable monitoring indicators that can prompt early intervention, ultimately improving survival outcomes and reducing mortality risk in hypertensive patients. The Cox regression analyses in the overall hypertensive population revealed that a higher antioxidant status (as indicated by a raised OBS) was correlated with a lower mortality risk. This is similar to previous studies that have focused solely on investigating the association between OBS and mortality risk (44). Building on this foundation, this study is the first to stratify the hypertensive population based on different hypertension control cascade outcomes to further examine the correlation between OBS and outcomes, while also identifying blood pressure control strategies that could enhance the mortality risk reduction associated with high OBS levels. Additionally, we explored the mediating roles of low-grade systemic inflammation and various organ functions in this association.

A key divergence we observed from previous studies was the distinct roles of the OBS subcomponents. Specifically, a favorable Nutrition-OBS was predominantly associated with reduced all-cause mortality, whereas a favorable Lifestyle-OBS showed a stronger association with reduced cardiovascular mortality. This distinction is pathophysiologically plausible and may reflect their different mechanisms of action. The robust association between a favorable Lifestyle-OBS and reduced cardiovascular mortality is expected, as its components—physical activity, BMI, smoking, and alcohol—are established primary risk factors for cardiovascular disease (45). They directly target the cardiovascular system by modulating key pathological processes such as endothelial dysfunction, atherosclerosis, and blood pressure regulation (46). Their potent and targeted effects on vascular health likely account for their pronounced impact on cardiovascular-specific mortality.

In contrast, the Nutrition-OBS encompasses a broad spectrum of micronutrients and dietary factors that exert more systemic and pleiotropic effects. These components are fundamental to maintaining cellular integrity, supporting immune surveillance, and ensuring proper DNA repair (47). These processes are critical for mitigating risks of various chronic diseases, including cancer, and are key determinants of healthy aging and overall longevity, thus impacting all-cause mortality (48). Importantly, our analysis found no synergistic interaction between these two subscores, suggesting that improvements in diet and lifestyle offer independent and additive benefits for survival.

Oxidative stress is characterized by an excessive buildup of reactive oxygen species (ROS) within the body, surpassing the capacity of the antioxidant system to neutralize them, ultimately leading to cellular and tissue damage (49). An elevated antioxidant status could potentially be associated with reduced ROS levels, thereby lowering mortality risk among hypertensive individuals from the initiation of hypertension. Oxidative stress contributes to hypertension not only by directly damaging vascular endothelium and inhibiting nitric oxide (NO) production but also by exacerbating hypertension progression through vascular remodeling and other pathways (19). Blood pressure control status serves as a critical marker of hypertension progression. The sustained adherence to a lifestyle defined by high antioxidant levels may exert antihypertensive effects during the maintenance and progression of hypertension through multiple mechanisms, including the attenuation of oxidative stress, enhancement of redox homeostasis, reduction of inflammation, and increased bioavailability of nitric oxide (NO) (31, 50, 51). Given the interplay between oxidative stress levels and the maintenance and progression of hypertension, we performed subgroup analyses to investigate the interaction between blood pressure control status and OBS scores, as well as their impact on mortality risk differences. The findings from the interaction analysis indicated that the distribution of OBS scores differed among patients with varying blood pressure control statuses. Well-controlled blood pressure (controlled hypertension) was generally associated with higher antioxidant levels, which also correlated with lower mortality risk. Among hypertensive individuals with similarly elevated antioxidant levels, those with controlled hypertension exhibited a 57% lower risk of all-cause mortality, whereas those with uncontrolled hypertension demonstrated only an 18% risk reduction (*P* for interaction = 0.017). Furthermore, the risk of cardiovascular mortality decreased by 71% in patients with controlled hypertension, compared to a modest 20% reduction in those with uncontrolled hypertension (*P* for interaction = 0.026). A well-controlled blood pressure status can amplify the benefits of an antioxidant lifestyle, possibly because blood pressure regulation reduces the baseline burden of oxidative stress and inflammation, thereby allowing antioxidant interventions to more effectively improve vascular function and redox homeostasis (31, 50, 52). To strengthen the reliability and validity of our findings and investigate the detailed effects of OBS on mortality risk within the hypertension control cascade, we performed sensitivity analyses. In sensitivity analyses, we excluded participants with well-controlled hypertension and stratified uncontrolled hypertensive individuals based on hypertension control cascade outcomes, conducting both subgroup analyses and interaction tests (*P* for interaction = 0.025). An analysis utilizing NHANES 2017–2020 data evaluated the response of the hypertension control cascade in U.S. adults with uncontrolled hypertension after recent updates to clinical guidelines, yielding baseline findings similar to our study (38). Our study also applied weighted data to represent the U.S. hypertensive population, ensuring that the sensitivity analysis results provide tailored intervention strategies and tendencies for patients with unsatisfactory blood pressure control, with the ultimate goal of enhancing antioxidant levels or OBS scores to reduce mortality risk. As previously mentioned, the hypertension control cascade encompasses three key components: awareness, treatment, and control (3, 8). Compared to low levels of antioxidant exposure, the mortality risk reduction associated with higher antioxidant levels differed across hypertension control cascade subgroups. Among individuals unaware of their hypertension, mortality risk was reduced by only 10%, whereas in those who were aware of their condition and met lifestyle modification and pharmacological treatment criteria, the reduction reached 40%. Furthermore, among patients who were currently taking antihypertensive medication but had poor blood pressure control, mortality risk was reduced by 46%. These findings underscore the necessity of identifying unrecognized hypertensive individuals at the primary care level, establishing adherence and adaptability assessment systems for treatment recommendations, and ultimately ensuring that patients aware of their hypertension and eligible for pharmacological treatment receive appropriate antihypertensive therapy with proper efficacy monitoring. Facilitating the transition of uncontrolled hypertensive individuals to a well-controlled hypertension status may further enhance the benefits of an antioxidant-rich lifestyle in reducing mortality risk.

Although the previous section examined the impact of oxidative stress on mortality outcomes, in actual clinical practice, the mortality of hypertensive patients is often linked to dysfunction in single or multiple organs or systems, resulting from persistent oxidative stress. Oxidative stress contributes to multi-organ damage through mechanisms such as vascular endothelial injury, vascular remodeling, structural alterations in organs such as the heart and kidneys, and the promotion of immune responses (15, 53–55). As inflammatory responses intensify and organ dysfunction progresses, hypertensive individuals face severe challenges to their quality of life, ultimately leading to increased mortality risk (56). However, within hypertensive populations, there remains no consensus regarding which specific organs or system function indicators should be prioritized for individuals exhibiting elevated oxidative stress levels. Therefore, investigating the mediating role of OBS in clinical outcomes is crucial for identifying early biomarkers of organ dysfunction in hypertensive patients and exploring the differential contributions of various organs. Our results demonstrated that both eGFR and UACR mediated the association between hypertension and mortality. This aligns with findings from a systematic review by Hernández-Ruiz et al. (2019), which noted that the validation of OBS often relies on its association with downstream biomarkers of inflammation and organ function when direct measures of oxidative stress are unavailable. This suggests that eGFR and UACR not only serve as essential markers for renal function assessment but also represent potential biomarkers of oxidative stress-induced kidney damage in hypertension, offering mechanistic insights into its pathophysiological impact (57). With persistent oxidative stress, a substantial proportion of hypertensive patients develop chronic kidney disease (CKD). Thus, maintaining optimal kidney function may serve as an effective strategy to mitigate the progression toward mortality in hypertensive patients (58). Additionally, a proportion of the mediation effect was attributed to serum uric acid (SUA). EThe limitations of this study include its retrospective xisting studies have shown that elevated uric acid levels increase susceptibility to oxidative stress and are closely associated with pro-oxidant and pro-inflammatory states. Lowering uric acid levels has been proposed to enhance patient outcomes and overall quality of life (59). To achieve a comprehensive assessment of systemic inflammation, we selected NLR and SIRI as composite inflammatory indices. Compared with absolute blood cell counts, NLR and SIRI more effectively reflect systemic inflammatory status (39). Our findings further revealed that NLR and SIRI exhibited significant mediation effects, reinforcing the critical role of inflammation control in improving patient prognosis. Low levels of systemic inflammation generally indicate early-stage pathophysiological alterations. However, as inflammation progresses, both structural and functional changes in organs and systems occur, leading to adverse inflammatory outcomes such as fibrosis and subsequent organ dysfunction (21, 60). Furthermore, liver-related markers did not exhibit significant mediation effects, likely due to the fact that oxidative stress in hypertension primarily affects vascular structures and organs directly influenced by blood pressure fluctuations, such as the heart and kidneys. In contrast, the liver is often secondarily influenced within the framework of systemic inflammation and oxidative stress, potentially through its role in immune regulation and metabolic function (61, 62). Lastly, as the NHANES database lacks precise indicators for the cardiac and neurological function required for mediation analysis, we performed an interaction analysis using self-reported histories of CVD and stroke as proxies. The interaction analysis between OBS and a history of cardiovascular events or stroke yielded negative results. This may be attributed to the role of oxidative stress as an initiating factor in the impairment of cardiac function and the nervous system. Through mechanisms such as sympathetic nervous system activation, vascular endothelial damage, increased cardiac workload, and accelerated atherosclerosis, oxidative stress profoundly disrupts the normal functioning of the heart and nervous system (63).

Our findings have several potential translational and public health implications. The OBS, as a composite measure of diet and lifestyle, could be developed into a simple, non-invasive screening tool for clinical practice. It could help clinicians identify high-risk hypertensive individuals with a pro-oxidant lifestyle and diet, thereby facilitating targeted counseling and personalized intervention strategies. From a public health perspective, our results, particularly the additive effects of the nutrition and lifestyle subscores, underscore the importance of dual-pronged health promotion campaigns. These campaigns should advocate simultaneously for antioxidant-rich dietary patterns and healthy lifestyle behaviors, such as smoking cessation and weight management.

However, the practical implementation of the OBS requires further research. Future studies should focus on developing and validating a simplified version of the score for routine clinical use. Ultimately, randomized controlled trials are needed to determine whether interventions designed to actively improve an individual's OBS can causally lead to a tangible reduction in mortality and cardiovascular events.

A primary limitation of our study is the lack of direct biomarkers of oxidative stress; instead, we relied on the OBS as a validated proxy score. This is a common challenge in large-scale epidemiological studies. Indeed, a comprehensive review by Hernández-Ruiz et al. (2019) highlighted this as a field-wide issue, noting that among 21 published OBS studies, only three had been validated against direct OS-related biomarkers. Other limitations include the retrospective design, which only represents the U.S. population, potentially restricting the applicability of these findings to broader populations. Additionally, due to data constraints, hypertension could not be classified as primary or secondary, which may introduce heterogeneity in the analysis. Furthermore, the OBS was assessed only at baseline, which does not account for changes over the follow-up period, and despite extensive covariate adjustment, residual confounding from unmeasured factors such as medication adherence, psychological stress, and chronic disease duration cannot be excluded. Additional clinical investigations are required to clarify whether cardiac and neurological dysfunction in hypertension serves as a mediating effect of oxidative stress in disease progression. It is also important to note that our mediation analysis relies on the key assumption of no unmeasured confounding, which may not be fully met in an observational setting. Therefore, these mediation results should be interpreted as exploratory and hypothesis-generating.”

## 5 Conclusions

Our findings indicate that elevated OBS levels are linked to a lower risk of both all-cause and cardiovascular mortality across different hypertension control cascade subgroups. This association was more pronounced in individuals with controlled hypertension. These findings highlight the importance of incorporating the hypertension control cascade into patient management, as it may enhance the mortality risk reduction benefits associated with higher OBS levels. Additionally, low-grade systemic inflammatory markers, eGFR, UACR, and serum uric acid may serve as early indicators of organ dysfunction in hypertensive patients, offering valuable insights into the potential mechanisms by which OBS contributes to reduced mortality risk in hypertension.

## Data Availability

The original contributions presented in the study are included in the article/Supplementary Material, further inquiries can be directed to the corresponding author.
