# Supplementary material for: The role of oxidative stress in modulating mortality risk across the hypertension control cascade
Source: Front Cardiovasc Med. 2025 Sep 8;12:1621911. doi: 10.3389/fcvm.2025.1621911 (PMC12450914; doi:10.3389/fcvm.2025.1621911)
Supplement: Supplementary file 1 [file Datasheet1.docx]

**Supplementary Table 1 Assignment scheme for oxidative balance score (OBS) based on antioxidant and prooxidant components among U.S. adults with hypertension (N = 9,305), stratified by sex. Each component was scored from 0 to 2 based on sex-specific tertiles.**

|  | **Property** |  | **Male** |  |  | **Female** |  |
| --- | --- | --- | --- | --- | --- | --- | --- |
| **OBS Components** | **Property** | **Male (0)** | **Male (1)** | **Male (2)** | **Female (0)** | **Female (1)** | **Female (2)** |
| Dietary fiber (g/d) | A | <12.7 | 12.7 - 20.7 | ≥20.7 | <10.7 | 10.7 - 17.4 | ≥17.4 |
| Carotene (RE/d) | A | <84.92 | 84.92 - 311.58 | ≥311.58 | <83.95 | 83.95 - 334.36 | ≥334.36 |
| Riboflavin (mg/d) | A | <1.68 | 1.68 - 2.59 | ≥2.59 | <1.32 | 1.32 - 1.97 | ≥1.97 |
| Niacin (mg/d) | A | <20.57 | 20.57 - 31.1 | ≥31.1 | <14.56 | 14.56 - 22.17 | ≥22.17 |
| Vitamin B6 (mg/d) | A | <1.59 | 1.59 - 2.47 | ≥2.47 | <1.17 | 1.17 - 1.81 | ≥1.81 |
| Total folate (mcg/d) | A | <311 | 311 - 491 | ≥491 | <241 | 241 - 381 | ≥381 |
| Vitamin B12 (mcg/d) | A | <3.13 | 3.13 - 6.04 | ≥6.04 | <2.18 | 2.18 - 4.21 | ≥4.21 |
| Vitamin C (mg/d) | A | <36.2 | 36.2 - 107.33 | ≥107.33 | <35.8 | 35.8 - 98.61 | ≥98.61 |
| Vitamin E (ATE) (mg/d) | A | <5.54 | 5.54 - 9.56 | ≥9.56 | <4.52 | 4.52 - 7.73 | ≥7.73 |
| Calcium (mg/d) | A | <644 | 644 - 1087 | ≥1087 | <540 | 540 - 886.33 | ≥886.33 |
| Zinc (mg/d) | A | <9.06 | 9.06 - 14.13 | ≥14.13 | <6.65 | 6.65 - 10.27 | ≥10.27 |
| Copper (mg/d) | A | <1.03 | 1.03 - 1.54 | ≥1.54 | <0.83 | 0.83 - 1.23 | ≥1.23 |
| Selenium (mcg/d) | A | <93.5 | 93.5 - 140.7 | ≥140.7 | <67.87 | 67.87 - 102.9 | ≥102.9 |
| Magnesium (mg/d) | A | <249.17 | 249.17 - 367 | ≥367 | <198 | 198 - 290 | ≥290 |
| Physical activity (MET-minute/week) | A | <600 | 600 - 1642.28 | ≥1642.28 | <427 | 427 - 1080 | ≥1080 |
| Total fat (g/d) | P | ≥101.7 | 64.82 - 101.7 | <64.82 | ≥74.92 | 46.98 - 74.92 | <46.98 |
| Iron (mg/d) | P | ≥18.35 | 11.97 - 18.35 | <11.97 | ≥13.95 | 9.09 - 13.95 | <9.09 |
| Body mass index (kg/m2) | P | ≥30.6 | 26.51 - 30.6 | <26.51 | ≥32.05 | 26.2 - 32.05 | <26.2 |
| Cotinine (ng/mL) | P | ≥0.28 | 0.03 - 0.28 | <0.03 | ≥0.07 | 0.02 - 0.07 | <0.02 |
| Alcohol (g/d) | P | ≥0.16 | 0 - 0.16 | <0 | ≥0 | 0 - 0 | <0 |

OBS: oxidative balance score; A: antioxidant; P: prooxidant; Q1 and Q3 represent the first and third tertiles, respectively. The OBS is composed of 20 elements, including 16 nutrient variables and 4 lifestyle variables, further classified into 5 pro-oxidant and 15 antioxidant components. Dietary information, encompassing key nutrients such as dietary fiber, carotenoids (expressed as retinol equivalents), antioxidant vitamins (e.g., α-tocopherol equivalents), and minerals (e.g., calcium, magnesium), was obtained through initial 24-hour dietary recall interviews. Lifestyle-associated determinants evaluated included physical activity levels, body mass index (BMI), alcohol consumption, and smoking habits, with smoking exposure quantified through serum cotinine concentrations. Within this framework, BMI, total fat intake, iron levels, alcohol intake, and smoking were categorized as pro-oxidative elements; all other 15 factors were designated as contributors to antioxidant capacity. Consistent with methods described previously, alcohol intake was categorized into three distinct levels: non-drinkers (score = 2), light-to-moderate drinkers (women: 0–15 g/day ; men: 0–30 g/day , score = 1), and heavy drinkers (women: ≥15 g/day ; men: ≥30 g/day , score = 0). The remaining 19 components were assigned scores (0, 1, or 2) according to tertiles stratified by sex. For antioxidant components, scores of 0, 1, and 2 were allocated corresponding respectively to the lowest, intermediate, and highest sex-specific tertiles of intake/level. For pro-oxidant components, this scoring system was applied inversely. The total OBS for each participant was computed by aggregating the individual scores from all 20 components, with higher total OBS scores signifying a greater predominance of antioxidant exposures relative to pro-oxidant exposures.

**Supplementary Table 2 Sensitivity analysis: Multivariable Cox regression of** **Nutrition-OBS and all-cause and cardiovascular mortality in U.S. adults with hypertension (N = 9,305 and 8,100).**

|  | **Model 1** | | | | | | **Model 2** | | | | | | **Model 3** | | | | | |
| --- | --- | --- | --- | --- | --- | --- | --- | --- | --- | --- | --- | --- | --- | --- | --- | --- | --- | --- |
| **Characteristic** | **HR**^1^ | **95% CI**^1^ | **p-value** |  | **p for trend** |  | **HR**^1^ | **95% CI**^1^ | **p-value** |  | **p for trend** |  | **HR**^1^ | **95% CI**^1^ | **p-value** |  | **p for trend** |  |
| All-cause death  Nutrition-OBS  (Quartile) | 0.97 | 0.96, 0.98 | <0.001 |  | <0.001 |  | 0.97 | 0.96, 0.98 | <0.001 |  | <0.001 |  | 0.98 | 0.97, 0.99 | <0.001 |  | <0.001 |  |
| Q1 | Reference | Reference |  |  |  |  | Reference | Reference |  |  |  |  | Reference | Reference |  |  |  |  |
| Q2 | 0.89 | 0.75, 1.05 | 0.2 |  |  |  | 0.88 | 0.74, 1.04 | 0.14 |  |  |  | 0.98 | 0.83, 1.16 | 0.8 |  |  |  |
| Q3 | 0.73 | 0.63, 0.84 | <0.001 |  |  |  | 0.72 | 0.62, 0.83 | <0.001 |  |  |  | 0.83 | 0.73, 0.95 | 0.008 |  |  |  |
| Q4 | 0.55 | 0.45, 0.68 | <0.001 |  |  |  | 0.55 | 0.45, 0.68 | <0.001 |  |  |  | 0.69 | 0.56, 0.86 | <0.001 |  |  |  |
| Cardiovascular death |  |  |  |  | <0.001 |  |  |  |  |  | <0.001 |  |  |  |  |  | 0.012 |  |
| Nutrition-OBS  (Quartile) | 0.96 | 0.94, 0.98 | <0.001 |  |  |  | 0.96 | 0.94, 0.98 | <0.001 |  |  |  | 0.97 | 0.95, 0.99 | 0.012 |  |  |  |
| Q1 | Reference | Reference |  |  |  |  | Reference | Reference |  |  |  |  | Reference | Reference |  |  |  |  |
| Q2 | 0.83 | 0.60, 1.14 | 0.2 |  |  |  | 0.79 | 0.57, 1.08 | 0.14 |  |  |  | 0.89 | 0.63, 1.25 | 0.5 |  |  |  |
| Q3 | 0.58 | 0.42, 0.79 | <0.001 |  |  |  | 0.55 | 0.40, 0.76 | <0.001 |  |  |  | 0.69 | 0.50, 0.95 | 0.025 |  |  |  |
| Q4 | 0.52 | 0.33, 0.83 | 0.006 |  |  |  | 0.51 | 0.32, 0.81 | 0.004 |  |  |  | 0.66 | 0.41, 1.08 | 0.10 |  |  |  |

Model 1 adjusted for age. Model 2 adjusted for sex, age, and race/ethnicity. Model 3 adjusted for sex, age, race/ethnicity, educational level, marital status, poverty-to-income ratio, smoking history, alcohol consumption, health insurance status, healthcare utilization, and history of diabetes.

Abbreviation: HR, hazard ratio.

**Supplementary Table 3 Sensitivity analysis: Multivariable Cox regression of** **Lifestyle-OBS and all-cause and cardiovascular mortality in U.S. adults with hypertension (N = 9,305 and 8,100).**

|  | **Model 1** | | | | | | **Model 2** | | | | | | **Model 3** | | | | | |
| --- | --- | --- | --- | --- | --- | --- | --- | --- | --- | --- | --- | --- | --- | --- | --- | --- | --- | --- |
| **Characteristic** | **HR**^1^ | **95% CI**^1^ | **p-value** |  | **p for trend** |  | **HR**^1^ | **95% CI**^1^ | **p-value** |  | **p for trend** |  | **HR**^1^ | **95% CI**^1^ | **p-value** |  | **p for trend** |  |
| All-cause death  Lifestyle-OBS  (Quartile) | 0.92 | 0.89, 0.96 | <0.001 |  | <0.001 |  | 0.93 | 0.90, 0.97 | <0.001 |  | <0.001 |  | 0.98 | 0.94, 1.01 | 0.200 |  | 0.200 |  |
| Q1 | Reference | Reference |  |  |  |  | Reference | Reference |  |  |  |  | Reference | Reference |  |  |  |  |
| Q2 | 0.83 | 0.66, 1.06 | 0.14 |  |  |  | 0.86 | 0.67, 1.10 | 0.2 |  |  |  | 0.91 | 0.72, 1.15 | 0.4 |  |  |  |
| Q3 | 0.74 | 0.59, 0.95 | 0.016 |  |  |  | 0.77 | 0.60, 0.98 | 0.033 |  |  |  | 0.89 | 0.70, 1.14 | 0.4 |  |  |  |
| Q4 | 0.62 | 0.49, 0.79 | <0.001 |  |  |  | 0.67 | 0.52, 0.86 | 0.001 |  |  |  | 0.84 | 0.65, 1.08 | 0.2 |  |  |  |
| Cardiovascular death |  |  |  |  | <0.001 |  |  |  |  |  | <0.001 |  |  |  |  |  | 0.017 |  |
| Lifestyle-OBS  (Quartile) | 0.86 | 0.80, 0.92 | <0.001 |  |  |  | 0.88 | 0.82, 0.94 | <0.001 |  |  |  | 0.92 | 0.85, 0.98 | 0.017 |  |  |  |
| Q1 | Reference | Reference |  |  |  |  | Reference | Reference |  |  |  |  | Reference | Reference |  |  |  |  |
| Q2 | 0.62 | 0.39, 0.98 | 0.041 |  |  |  | 0.66 | 0.41, 1.06 | 0.086 |  |  |  | 0.69 | 0.44, 1.10 | 0.12 |  |  |  |
| Q3 | 0.56 | 0.38, 0.84 | 0.005 |  |  |  | 0.61 | 0.40, 0.91 | 0.016 |  |  |  | 0.69 | 0.46, 1.05 | 0.085 |  |  |  |
| Q4 | 0.37 | 0.23, 0.57 | <0.001 |  |  |  | 0.43 | 0.27, 0.67 | <0.001 |  |  |  | 0.55 | 0.34, 0.89 | 0.014 |  |  |  |

Model 1 adjusted for age. Model 2 adjusted for sex, age, and race/ethnicity. Model 3 adjusted for sex, age, race/ethnicity, educational level, marital status, poverty-to-income ratio, smoking history, alcohol consumption, health insurance status, healthcare utilization, and history of diabetes.

Abbreviation: HR, hazard ratio.

**Supplementary Table 4 Stratified analysis of the association between oxidative balance score (OBS) and mortality in U.S. adults with hypertension (N = 9,305).**

| Variable | Count | Percent | Levels | Point Estimate | Lower | Upper | P value | P for interaction | FDR-adjusted q-value |
| --- | --- | --- | --- | --- | --- | --- | --- | --- | --- |
| Age |  |  |  |  |  |  |  | 0.575 | 0.903 |
| 18-44 | 2499 | 26.8 | Q1 | Reference |  |  |  |  |  |
|  |  |  | Q2 | 1.6 | 0.68 | 3.79 | 1.079 |  |  |
|  |  |  | Q3 | 0.77 | 0.31 | 1.89 | <0.001 |  |  |
|  |  |  | Q4 | 0.9 | 0.32 | 2.52 | <0.001 |  |  |
| 45-64 | 3690 | 39.6 | Q1 | Reference |  |  |  |  |  |
|  |  |  | Q2 | 0.88 | 0.64 | 1.22 | <0.001 |  |  |
|  |  |  | Q3 | 0.79 | 0.59 | 1.08 | <0.001 |  |  |
|  |  |  | Q4 | 0.34 | 0.22 | 0.52 | <0.001 |  |  |
| ≥65 | 3116 | 33.6 | Q1 | Reference |  |  |  |  |  |
|  |  |  | Q2 | 0.95 | 0.77 | 1.17 | <0.001 |  |  |
|  |  |  | Q3 | 0.82 | 0.68 | 0.99 | <0.001 |  |  |
|  |  |  | Q4 | 0.68 | 0.55 | 0.83 | <0.001 |  |  |
| Gender |  |  |  |  |  |  |  | 0.354 | 0.826 |
| male | 5396 | 57.9 | Q1 | Reference |  |  |  |  |  |
|  |  |  | Q2 | 1.01 | 0.79 | 1.29 | 0.091 |  |  |
|  |  |  | Q3 | 0.83 | 0.66 | 1.05 | <0.001 |  |  |
|  |  |  | Q4 | 0.54 | 0.41 | 0.7 | <0.001 |  |  |
| female | 3909 | 42.1 | Q1 | Reference |  |  |  |  |  |
|  |  |  | Q2 | 1.08 | 0.82 | 1.42 | 0.522 |  |  |
|  |  |  | Q3 | 0.98 | 0.76 | 1.27 | <0.001 |  |  |
|  |  |  | Q4 | 0.65 | 0.49 | 0.87 | <0.001 |  |  |
| Race/ethnicity |  |  |  |  |  |  |  | 0.102 | 0.476 |
| Mexican American | 1146 | 12.3 | Q1 | Reference |  |  |  |  |  |
|  |  |  | Q2 | 0.78 | 0.45 | 1.37 | <0.001 |  |  |
|  |  |  | Q3 | 0.52 | 0.28 | 0.96 | <0.001 |  |  |
|  |  |  | Q4 | 0.28 | 0.12 | 0.62 | <0.001 |  |  |
| Other Hispanic | 552 | 5.9 | Q1 | Reference |  |  |  |  |  |
|  |  |  | Q2 | 0.96 | 0.49 | 1.88 | <0.001 |  |  |
|  |  |  | Q3 | 0.71 | 0.28 | 1.79 | <0.001 |  |  |
|  |  |  | Q4 | 0.47 | 0.22 | 1.02 | <0.001 |  |  |
| Non-Hispanic White | 4664 | 50.2 | Q1 | Reference |  |  |  |  |  |
|  |  |  | Q2 | 1.08 | 0.87 | 1.34 | 0.727 |  |  |
|  |  |  | Q3 | 0.92 | 0.77 | 1.1 | <0.001 |  |  |
|  |  |  | Q4 | 0.57 | 0.46 | 0.71 | <0.001 |  |  |
| Non-Hispanic Black | 2122 | 22.8 | Q1 | Reference |  |  |  |  |  |
|  |  |  | Q2 | 0.7 | 0.43 | 1.13 | <0.001 |  |  |
|  |  |  | Q3 | 0.67 | 0.44 | 1.01 | <0.001 |  |  |
|  |  |  | Q4 | 0.68 | 0.41 | 1.14 | <0.001 |  |  |
| Other Race | 821 | 8.8 | Q1 | Reference |  |  |  |  |  |
|  |  |  | Q2 | 1.32 | 0.56 | 3.12 | 0.628 |  |  |
|  |  |  | Q3 | 0.67 | 0.24 | 1.89 | <0.001 |  |  |
|  |  |  | Q4 | 0.89 | 0.34 | 2.31 | <0.001 |  |  |
| Marital status |  |  |  |  |  |  |  | 0.892 | 0.903 |
| married | 5414 | 58.1 | Q1 | Reference |  |  |  |  |  |
|  |  |  | Q2 | 1.1 | 0.88 | 1.38 | 0.82 |  |  |
|  |  |  | Q3 | 0.87 | 0.7 | 1.07 | <0.001 |  |  |
|  |  |  | Q4 | 0.56 | 0.45 | 0.71 | <0.001 |  |  |
| single | 3410 | 36.8 | Q1 | Reference |  |  |  |  |  |
|  |  |  | Q2 | 1.03 | 0.77 | 1.37 | 0.176 |  |  |
|  |  |  | Q3 | 1.06 | 0.77 | 1.44 | 0.343 |  |  |
|  |  |  | Q4 | 0.66 | 0.47 | 0.92 | <0.001 |  |  |
| living with partner | 481 | 5.2 | Q1 | Reference |  |  |  |  |  |
|  |  |  | Q2 | 0.61 | 0.18 | 2.07 | <0.001 |  |  |
|  |  |  | Q3 | 0.17 | 0.04 | 0.69 | <0.001 |  |  |
|  |  |  | Q4 | 0.49 | 0.13 | 1.85 | <0.001 |  |  |
| Educational attainment |  |  |  |  |  |  |  | 0.665 | 0.903 |
| Less than high school | 1733 | 18.6 | Q1 | Reference |  |  |  |  |  |
|  |  |  | Q2 | 0.85 | 0.6 | 1.21 | <0.001 |  |  |
|  |  |  | Q3 | 0.76 | 0.54 | 1.07 | <0.001 |  |  |
|  |  |  | Q4 | 0.62 | 0.42 | 0.92 | <0.001 |  |  |
| High school graduate, general educational development, or equivalent | 2151 | 23.1 | Q1 | Reference |  |  |  |  |  |
|  |  |  | Q2 | 1.12 | 0.79 | 1.58 | 0.632 |  |  |
|  |  |  | Q3 | 1.01 | 0.72 | 1.43 | 0.082 |  |  |
|  |  |  | Q4 | 0.69 | 0.47 | 1.03 | <0.001 |  |  |
| Some college or associates degree | 2864 | 30.9 | Q1 | Reference |  |  |  |  |  |
|  |  |  | Q2 | 1.49 | 1.04 | 2.14 | 2.155 |  |  |
|  |  |  | Q3 | 1.51 | 1.09 | 2.09 | 2.455 |  |  |
|  |  |  | Q4 | 0.83 | 0.55 | 1.26 | <0.001 |  |  |
| College graduate or above | 2557 | 27.4 | Q1 | Reference |  |  |  |  |  |
|  |  |  | Q2 | 0.91 | 0.61 | 1.36 | <0.001 |  |  |
|  |  |  | Q3 | 0.66 | 0.43 | 1.02 | <0.001 |  |  |
|  |  |  | Q4 | 0.55 | 0.36 | 0.83 | <0.001 |  |  |
| PIR |  |  |  |  |  |  |  | 0.774 | 0.903 |
| ≤1.30 | 2074 | 22.2 | Q1 | Reference |  |  |  |  |  |
|  |  |  | Q2 | 0.69 | 0.49 | 0.97 | <0.001 |  |  |
|  |  |  | Q3 | 0.55 | 0.36 | 0.86 | <0.001 |  |  |
|  |  |  | Q4 | 0.61 | 0.41 | 0.91 | <0.001 |  |  |
| >1.30 and ≤3.50 | 3465 | 37.4 | Q1 | Reference |  |  |  |  |  |
|  |  |  | Q2 | 1.12 | 0.88 | 1.42 | 0.899 |  |  |
|  |  |  | Q3 | 1 | 0.74 | 1.36 | 0.03 |  |  |
|  |  |  | Q4 | 0.75 | 0.56 | 1.02 | <0.001 |  |  |
| ＞3.50 | 3766 | 40.4 | Q1 | Reference |  |  |  |  |  |
|  |  |  | Q2 | 1.45 | 1.06 | 1.99 | 2.311 |  |  |
|  |  |  | Q3 | 1.21 | 0.9 | 1.64 | 1.257 |  |  |
|  |  |  | Q4 | 0.66 | 0.47 | 0.93 | <0.001 |  |  |
| Drinking |  |  |  |  |  |  |  | 0.038 | 0.469 |
| Yes | 6877 | 73.8 | Q1 | Reference |  |  |  |  |  |
|  |  |  | Q2 | 1.02 | 0.83 | 1.26 | 0.19 |  |  |
|  |  |  | Q3 | 0.88 | 0.71 | 1.09 | <0.001 |  |  |
|  |  |  | Q4 | 0.54 | 0.43 | 0.68 | <0.001 |  |  |
| No | 2428 | 26.2 | Q1 | Reference |  |  |  |  |  |
|  |  |  | Q2 | 1.09 | 0.8 | 1.48 | 0.531 |  |  |
|  |  |  | Q3 | 0.98 | 0.72 | 1.33 | <0.001 |  |  |
|  |  |  | Q4 | 0.78 | 0.59 | 1.04 | <0.001 |  |  |
| Health insurance |  |  |  |  |  |  |  | 0.206 | 0.721 |
| Insured | 8097 | 86.8 | Q1 | Reference |  |  |  |  |  |
|  |  |  | Q2 | 1.03 | 0.85 | 1.24 | 0.265 |  |  |
|  |  |  | Q3 | 0.87 | 0.73 | 1.03 | <0.001 |  |  |
|  |  |  | Q4 | 0.55 | 0.45 | 0.68 | <0.001 |  |  |
| Uninsured | 1208 | 13.2 | Q1 | Reference |  |  |  |  |  |
|  |  |  | Q2 | 0.9 | 0.41 | 1.98 | <0.001 |  |  |
|  |  |  | Q3 | 1.04 | 0.52 | 2.08 | 0.105 |  |  |
|  |  |  | Q4 | 0.91 | 0.43 | 1.91 | <0.001 |  |  |
| Healthcare |  |  |  |  |  |  |  | 0.28 | 0.784 |
| 0 | 1104 | 11.8 | Q1 | Reference |  |  |  |  |  |
|  |  |  | Q2 | 2.26 | 1.23 | 4.14 | 2.619 |  |  |
|  |  |  | Q3 | 1.03 | 0.52 | 2.04 | 0.085 |  |  |
|  |  |  | Q4 | 0.8 | 0.33 | 1.94 | <0.001 |  |  |
| 1 | 1521 | 16.3 | Q1 | Reference |  |  |  |  |  |
|  |  |  | Q2 | 1.13 | 0.64 | 2.01 | 0.417 |  |  |
|  |  |  | Q3 | 1.3 | 0.78 | 2.15 | 1.003 |  |  |
|  |  |  | Q4 | 1.04 | 0.59 | 1.83 | 0.146 |  |  |
| ≥2 | 6680 | 71.8 | Q1 | Reference |  |  |  |  |  |
|  |  |  | Q2 | 0.92 | 0.78 | 1.09 | <0.001 |  |  |
|  |  |  | Q3 | 0.8 | 0.67 | 0.95 | <0.001 |  |  |
|  |  |  | Q4 | 0.5 | 0.41 | 0.6 | <0.001 |  |  |
| Smoking |  |  |  |  |  |  |  | 0.067 | 0.469 |
| No | 4991 | 53.5 | Q1 | Reference |  |  |  |  |  |
|  |  |  | Q2 | 1.41 | 1.05 | 1.87 | 2.323 |  |  |
|  |  |  | Q3 | 0.95 | 0.69 | 1.31 | <0.001 |  |  |
|  |  |  | Q4 | 0.76 | 0.55 | 1.06 | <0.001 |  |  |
| Yes | 4314 | 46.5 | Q1 | Reference |  |  |  |  |  |
|  |  |  | Q2 | 0.84 | 0.67 | 1.05 | <0.001 |  |  |
|  |  |  | Q3 | 0.87 | 0.7 | 1.08 | <0.001 |  |  |
|  |  |  | Q4 | 0.5 | 0.39 | 0.65 | <0.001 |  |  |
| Kidney function |  |  |  |  |  |  |  | 0.616 | 0.903 |
| GF-1 | 3502 | 37.6 | Q1 | Reference |  |  |  |  |  |
|  |  |  | Q2 | 1.06 | 0.7 | 1.61 | 0.29 |  |  |
|  |  |  | Q3 | 0.94 | 0.64 | 1.38 | <0.001 |  |  |
|  |  |  | Q4 | 0.64 | 0.43 | 0.97 | <0.001 |  |  |
| GF-2 | 4671 | 50.3 | Q1 | Reference |  |  |  |  |  |
|  |  |  | Q2 | 1.11 | 0.85 | 1.44 | 0.759 |  |  |
|  |  |  | Q3 | 0.92 | 0.72 | 1.19 | <0.001 |  |  |
|  |  |  | Q4 | 0.58 | 0.44 | 0.76 | <0.001 |  |  |
| GF-3 and above | 1132 | 12.1 | Q1 | Reference |  |  |  |  |  |
|  |  |  | Q2 | 0.92 | 0.65 | 1.31 | <0.001 |  |  |
|  |  |  | Q3 | 0.89 | 0.63 | 1.26 | <0.001 |  |  |
|  |  |  | Q4 | 0.73 | 0.47 | 1.14 | <0.001 |  |  |
| Diabetes |  |  |  |  |  |  |  | 0.806 | 0.903 |
| No | 7520 | 80.9 | Q1 | Reference |  |  |  |  |  |
|  |  |  | Q2 | 1.04 | 0.86 | 1.27 | 0.423 |  |  |
|  |  |  | Q3 | 0.89 | 0.75 | 1.06 | <0.001 |  |  |
|  |  |  | Q4 | 0.6 | 0.49 | 0.75 | <0.001 |  |  |
| Yes | 1785 | 19.1 | Q1 | Reference |  |  |  |  |  |
|  |  |  | Q2 | 1.04 | 0.74 | 1.47 | 0.246 |  |  |
|  |  |  | Q3 | 0.93 | 0.65 | 1.33 | <0.001 |  |  |
|  |  |  | Q4 | 0.66 | 0.45 | 0.96 | <0.001 |  |  |
| Stroke |  |  |  |  |  |  |  | 0.903 | 0.903 |
| Yes | 337 | 3.6 | Q1 | Reference |  |  |  |  |  |
|  |  |  | Q2 | 1.31 | 0.41 | 4.15 | 0.453 |  |  |
|  |  |  | Q3 | 0.91 | 0.27 | 3.07 | <0.001 |  |  |
|  |  |  | Q4 | 0.5 | 0.21 | 1.21 | <0.001 |  |  |
| No | 8968 | 96.4 | Q1 | Reference |  |  |  |  |  |
|  |  |  | Q2 | 1.18 | 0.87 | 1.61 | 1.053 |  |  |
|  |  |  | Q3 | 0.87 | 0.6 | 1.25 | <0.001 |  |  |
|  |  |  | Q4 | 0.7 | 0.44 | 1.12 | <0.001 |  |  |
| Cardiovascular disease |  |  |  |  |  |  |  | 0.655 | 0.903 |
| Yes | 992 | 10.7 | Q1 | Reference |  |  |  |  |  |
|  |  |  | Q2 | 1.06 | 0.65 | 1.74 | 0.246 |  |  |
|  |  |  | Q3 | 0.88 | 0.49 | 1.58 | <0.001 |  |  |
|  |  |  | Q4 | 0.5 | 0.26 | 0.95 | <0.001 |  |  |
| No | 8313 | 89.3 | Q1 | Reference |  |  |  |  |  |
|  |  |  | Q2 | 1.09 | 0.69 | 1.73 | 0.365 |  |  |
|  |  |  | Q3 | 0.78 | 0.48 | 1.24 | <0.001 |  |  |
|  |  |  | Q4 | 0.79 | 0.46 | 1.37 | <0.001 |  |  |
|  |  |  |  |  |  |  |  |  |  |

Adjusted for sex, age, race/ethnicity, educational level, marital status, poverty-to-income ratio, smoking history, alcohol consumption, health insurance status, healthcare utilization, and history of diabetes.

**Supplementary Table 5 Sensitivity Analysis: Associations of** **Nutrition-OBS Quartile Groups with All-Cause and Cardiovascular Mortality in U.S. Adults with Hypertension (All-cause death: N = 9,305; Cardiovascular death: N = 8,100).**

| Variable | | Count | Percent | | | Levels | Point Estimate | Lower | Upper | | P value | P for interaction | FDR-adjusted q-value |
| --- | --- | --- | --- | --- | --- | --- | --- | --- | --- | --- | --- | --- | --- |
| All-cause death | |  |  | | |  |  |  |  | |  |  |  |
| Hypertension Subgroups | | 9305 |  | | |  |  |  |  | |  | 0.101 | 0.147 |
| Controlled hypertension | | 1665 | 17.9 | | | Q1 | Reference |  |  | |  |  |  |
|  | |  |  | | | Q2 | 0.69 | 0.45 | 1.05 | | <0.001 |  |  |
|  | |  |  | | | Q3 | 0.58 | 0.4 | 0.86 | | <0.001 |  |  |
|  | |  |  | | | Q4 | 0.47 | 0.28 | 0.79 | | <0.001 |  |  |
| Uncontrolled hypertension | | 7640 | 82.1 | | | Q1 | Reference |  |  | |  |  |  |
|  | |  |  | | | Q2 | 1.07 | 0.89 | 1.28 | | 0.717 |  |  |
|  | |  |  | | | Q3 | 0.92 | 0.78 | 1.07 | | <0.001 |  |  |
|  | |  |  | | | Q4 | 0.76 | 0.61 | 0.96 | | <0.001 |  |  |
| Cardiovascular death |  | | |  |  | |  |  |  |  | |  |  |
| Hypertension Subgroups | 8100 | | |  |  | |  |  |  |  | | 0.052 | 0.147 |
| Controlled hypertension | 1442 | | | 17.8 | Q1 | | Reference |  |  |  | |  |  |
|  |  | | |  | Q2 | | 0.98 | 0.52 | 1.86 | <0.001 | |  |  |
|  |  | | |  | Q3 | | 0.49 | 0.24 | 1.03 | <0.001 | |  |  |
|  |  | | |  | Q4 | | 0.31 | 0.1 | 0.98 | <0.001 | |  |  |
| Uncontrolled hypertension | 6658 | | | 82.2 | Q1 | | Reference |  |  |  | |  |  |
|  |  | | |  | Q2 | | 0.92 | 0.61 | 1.41 | <0.001 | |  |  |
|  |  | | |  | Q3 | | 0.8 | 0.54 | 1.18 | <0.001 | |  |  |
|  |  | | |  | Q4 | | 0.85 | 0.48 | 1.5 | <0.001 | |  |  |

Adjusted for sex, age, race/ethnicity, educational level, marital status, poverty-to-income ratio, smoking history, alcohol consumption, health insurance status, healthcare utilization, and history of diabetes.

**Supplementary Table 6 Sensitivity Analysis: Associations of Lifestyle-OBS Quartile Groups with All-Cause and Cardiovascular Mortality in U.S. Adults with Hypertension (All-cause death: N = 9,305; Cardiovascular death: N = 8,100).**

| Variable | | Count | Percent | | | Levels | Point Estimate | Lower | Upper | | P value | P for interaction | FDR-adjusted q-value |
| --- | --- | --- | --- | --- | --- | --- | --- | --- | --- | --- | --- | --- | --- |
| All-cause death | |  |  | | |  |  |  |  | |  |  |  |
| Hypertension Subgroups | | 9305 |  | | |  |  |  |  | |  | 0.121 | 0.484 |
| Controlled hypertension | | 1665 | 17.9 | | | Q1 | Reference |  |  | |  |  |  |
|  | |  |  | | | Q2 | 1.33 | 0.78 | 2.26 | | 1.064 |  |  |
|  | |  |  | | | Q3 | 1.3 | 0.79 | 2.13 | | 1.015 |  |  |
|  | |  |  | | | Q4 | 1.48 | 0.8 | 2.74 | | 1.251 |  |  |
| Uncontrolled hypertension | | 7640 | 82.1 | | | Q1 | Reference |  |  | |  |  |  |
|  | |  |  | | | Q2 | 0.83 | 0.64 | 1.07 | | <0.001 |  |  |
|  | |  |  | | | Q3 | 0.81 | 0.62 | 1.06 | | <0.001 |  |  |
|  | |  |  | | | Q4 | 0.75 | 0.57 | 0.99 | | <0.001 |  |  |
| Cardiovascular death |  | | |  |  | |  |  |  |  | |  |  |
| Hypertension Subgroups | 8100 | | |  |  | |  |  |  |  | | 0.982 | 0.982 |
| Controlled hypertension | 1442 | | | 17.8 | Q1 | | Reference |  |  |  | |  |  |
|  |  | | |  | Q2 | | 0.55 | 0.24 | 1.28 | <0.001 | |  |  |
|  |  | | |  | Q3 | | 0.56 | 0.29 | 1.08 | <0.001 | |  |  |
|  |  | | |  | Q4 | | 0.58 | 0.22 | 1.53 | <0.001 | |  |  |
| Uncontrolled hypertension | 6658 | | | 82.2 | Q1 | | Reference |  |  |  | |  |  |
|  |  | | |  | Q2 | | 0.67 | 0.37 | 1.23 | <0.001 | |  |  |
|  |  | | |  | Q3 | | 0.67 | 0.39 | 1.17 | <0.001 | |  |  |
|  |  | | |  | Q4 | | 0.53 | 0.29 | 0.96 | <0.001 | |  |  |

Adjusted for sex, age, race/ethnicity, educational level, marital status, poverty-to-income ratio, smoking history, alcohol consumption, health insurance status, healthcare utilization, and history of diabetes.

**Supplementary Table 7 Sensitivity Analysis: Associations of Nutrition-OBS Quartile Groups with All-Cause and Cardiovascular Mortality After Excluding Participants with Controlled Hypertension and Stratifying into Four Hypertension Control Cascade Subgroups (All-cause death: N = 7,640; Cardiovascular death: N = 6,658).**

| Variable | Count | Percent | Levels | Point Estimate | Lower | Upper | P value | P for interaction | FDR-adjusted q-value |
| --- | --- | --- | --- | --- | --- | --- | --- | --- | --- |
| All-cause death |  |  |  |  |  |  |  |  |  |
| Hypertension Control Cascade Subgroups | 7640 |  |  |  |  |  |  | 0.259 | 0.259 |
| Unaware, not recommended treatment | 4027 | 52.7 | Q1 | Reference |  |  |  |  |  |
|  |  |  | Q2 | 1.36 | 0.98 | 1.87 | 1.856 |  |  |
|  |  |  | Q3 | 1 | 0.76 | 1.32 | 0.009 |  |  |
|  |  |  | Q4 | 0.94 | 0.61 | 1.45 | <0.001 |  |  |
| Aware, met criteria for lifestyle modifications | 651 | 8.5 | Q1 | Reference |  |  |  |  |  |
|  |  |  | Q2 | 1.36 | 0.98 | 1.87 | 1.856 |  |  |
|  |  |  | Q3 | 1 | 0.76 | 1.32 | 0.009 |  |  |
|  |  |  | Q4 | 0.94 | 0.61 | 1.45 | <0.001 |  |  |
| Aware, met criteria for lifestyle modifications and medication | 653 | 8.5 | Q1 | Reference |  |  |  |  |  |
|  |  |  | Q2 | - | - | - | - |  |  |
|  |  |  | Q3 | - | - | - | - |  |  |
|  |  |  | Q4 | - | - | - | - |  |  |
| Aware, met criteria for lifestyle modifications and medication,  and is currently taking BP medication | 2309 | 30.2 | Q1 | Reference |  |  |  |  |  |
|  |  |  | Q2 | 1.1 | 0.59 | 2.02 | 0.293 |  |  |
|  |  |  | Q3 | 0.71 | 0.38 | 1.32 | <0.001 |  |  |
|  |  |  | Q4 | 0.54 | 0.25 | 1.17 | <0.001 |  |  |
| Cardiovascular death |  |  |  |  |  |  |  |  |  |
| Hypertension Control Cascade Subgroups | 6658 |  |  |  |  |  |  | 0.110 | 0.147 |
| Unaware, not recommended treatment | 3621 | 54.4 | Q1 | Reference |  |  |  |  |  |
|  |  |  | Q2 | 1.25 | 0.71 | 2.2 | 0.763 |  |  |
|  |  |  | Q3 | 1.18 | 0.61 | 2.29 | 0.491 |  |  |
|  |  |  | Q4 | 1.67 | 0.84 | 3.29 | 1.474 |  |  |
| Aware, met criteria for lifestyle modifications | 631 | 9.5 | Q1 | Reference |  |  |  |  |  |
|  |  |  | Q2 | - | - | - | - |  |  |
|  |  |  | Q3 | - | - | - | - |  |  |
|  |  |  | Q4 | - | - | - | - |  |  |
| Aware, met criteria for lifestyle modifications and medication | 562 | 8.4 | Q1 | Reference |  |  |  |  |  |
|  |  |  | Q2 | 0.97 | 0.31 | 2.98 | <0.001 |  |  |
|  |  |  | Q3 | 0.31 | 0.04 | 2.13 | <0.001 |  |  |
|  |  |  | Q4 | 0.46 | 0.11 | 1.89 | <0.001 |  |  |
| Aware, met criteria for lifestyle modifications and medication,  and is currently taking BP medication | 1844 | 27.7 | Q1 | Reference |  |  |  |  |  |
|  |  |  | Q2 | 0.76 | 0.44 | 1.31 | <0.001 |  |  |
|  |  |  | Q3 | 0.7 | 0.41 | 1.18 | <0.001 |  |  |
|  |  |  | Q4 | 0.55 | 0.22 | 1.35 | <0.001 |  |  |

Adjusted for sex, age, race/ethnicity, educational level, marital status, poverty-to-income ratio, smoking history, alcohol consumption, health insurance status, healthcare utilization, and history of diabetes.

**Supplementary Table 8 Sensitivity Analysis: Associations of Lifestyle-OBS Quartile Groups with All-Cause and Cardiovascular Mortality After Excluding Participants with Controlled Hypertension and Stratifying into Four Hypertension Control Cascade Subgroups (All-cause death: N = 7,640; Cardiovascular death: N = 6,658).**

| Variable | Count | Percent | Levels | Point Estimate | Lower | Upper | P value | P for interaction | FDR-adjusted q-value |
| --- | --- | --- | --- | --- | --- | --- | --- | --- | --- |
| All-cause death |  |  |  |  |  |  |  |  |  |
| Hypertension Control Cascade Subgroups | 7640 |  |  |  |  |  |  | 0.974 | 0.982 |
| Unaware, not recommended treatment | 4027 | 52.7 | Q1 | Reference |  |  |  |  |  |
|  |  |  | Q2 | 0.7 | 0.46 | 1.06 | <0.001 |  |  |
|  |  |  | Q3 | 0.79 | 0.51 | 1.21 | <0.001 |  |  |
|  |  |  | Q4 | 0.79 | 0.51 | 1.25 | <0.001 |  |  |
| Aware, met criteria for lifestyle modifications | 651 | 8.5 | Q1 | Reference |  |  |  |  |  |
|  |  |  | Q2 | - | - | - | - |  |  |
|  |  |  | Q3 | - | - | - | - |  |  |
|  |  |  | Q4 | - | - | - | - |  |  |
| Aware, met criteria for lifestyle modifications and medication | 653 | 8.5 | Q1 | Reference |  |  |  |  |  |
|  |  |  | Q2 | 0.58 | 0.24 | 1.41 | <0.001 |  |  |
|  |  |  | Q3 | 0.56 | 0.27 | 1.16 | <0.001 |  |  |
|  |  |  | Q4 | 0.39 | 0.15 | 1.04 | <0.001 |  |  |
| Aware, met criteria for lifestyle modifications and medication,  and is currently taking BP medication | 2309 | 30.2 | Q1 | Reference |  |  |  |  |  |
|  |  |  | Q2 | 1.02 | 0.73 | 1.42 | 0.116 |  |  |
|  |  |  | Q3 | 0.9 | 0.65 | 1.25 | <0.001 |  |  |
|  |  |  | Q4 | 0.83 | 0.55 | 1.25 | <0.001 |  |  |
| Cardiovascular death |  |  |  |  |  |  |  |  |  |
| Hypertension Control Cascade Subgroups | 6658 |  |  |  |  |  |  | 0.514 | 0.982 |
| Unaware, not recommended treatment | 3621 | 54.4 | Q1 | Reference |  |  |  |  |  |
|  |  |  | Q2 | 0.56 | 0.2 | 1.52 | <0.001 |  |  |
|  |  |  | Q3 | 0.71 | 0.27 | 1.87 | <0.001 |  |  |
|  |  |  | Q4 | 0.36 | 0.13 | 1 | <0.001 |  |  |
| Aware, met criteria for lifestyle modifications | 631 | 9.5 | Q1 | Reference |  |  |  |  |  |
|  |  |  | Q2 | - | - | - | - |  |  |
|  |  |  | Q3 | - | - | - | - |  |  |
|  |  |  | Q4 | - | - | - | - |  |  |
| Aware, met criteria for lifestyle modifications and medication | 562 | 8.4 | Q1 | Reference |  |  |  |  |  |
|  |  |  | Q2 | 0.63 | 0.18 | 2.23 | <0.001 |  |  |
|  |  |  | Q3 | 0.78 | 0.12 | 5.17 | <0.001 |  |  |
|  |  |  | Q4 | 0.73 | 0.16 | 3.38 | <0.001 |  |  |
| Aware, met criteria for lifestyle modifications and medication,  and is currently taking BP medication | 1844 | 27.7 | Q1 | Reference |  |  |  |  |  |
|  |  |  | Q2 | 0.83 | 0.39 | 1.75 | <0.001 |  |  |
|  |  |  | Q3 | 0.7 | 0.34 | 1.43 | <0.001 |  |  |
|  |  |  | Q4 | 0.82 | 0.34 | 1.97 | <0.001 |  |  |

Adjusted for sex, age, race/ethnicity, educational level, marital status, poverty-to-income ratio, smoking history, alcohol consumption, health insurance status, healthcare utilization, and history of diabetes.

**Supplementary Table 9** **Sensitivity analysis: Exclusion of participants with missing variables and multivariable Cox regression of the association between oxidative balance score (OBS) and mortality in hypertensive adults (All-cause death: N = 9,305; Cardiovascular death: N = 8,100).**

|  | **Model 1** | | **Model 2** | | | | | | | | | | | | **Model 3** | | | | |
| --- | --- | --- | --- | --- | --- | --- | --- | --- | --- | --- | --- | --- | --- | --- | --- | --- | --- | --- | --- |
| **Characteristic** | **HR**^1^ | **95% CI**^1^ | **p-value** |  | **p for trend** |  | **HR**^1^ | **95% CI**^1^ | **p-value** |  | **p for trend** |  | **HR**^1^ | **95% CI**^1^ | | **p-value** |  | **p for trend** |  |
| All-cause death  OBS  OBS (Quartile) | 0.82 | 0.72, 0.92 | <0.001 |  | <0.001 |  | 0.82 | 0.72, 0.93 | =0.002 |  | <0.001 |  | 0.89 | 0.78, 0.97 | | =0.032 |  | <0.001 |  |
| Q1 | Reference | Reference |  |  |  |  | Reference | Reference |  |  |  |  | Reference | Reference | |  |  |  |  |
| Q2 | 0.98 | 0.71, 1.35 | 0.9 |  |  |  | 0.95 | 0.68, 1.31 | 0.7 |  |  |  | 1.09 | 0.78, 1.51 | | 0.6 |  |  |  |
| Q3 | 0.73 | 0.53, 1.00 | 0.053 |  |  |  | 0.71 | 0.51, 0.99 | 0.045 |  |  |  | 0.87 | 0.62, 0.87 | | 0.032 |  |  |  |
| Q4 | 0.55 | 0.36, 0.82 | 0.003 |  |  |  | 0.55 | 0.36, 0.83 | 0.005 |  |  |  | 0.71 | 0.45, 0.98 | | 0.007 |  |  |  |
| Cardiovascular death |  |  |  |  | <0.001 |  |  |  |  |  | <0.001 |  |  |  | |  |  | <0.001 |  |
| OBS  OBS (Quartile) | 0.83 | 0.79, 0.88 | <0.001 |  |  |  | 0.83 | 0.79, 0.88 | <0.001 |  |  |  | 0.90 | 0.85, 0.95 | | <0.001 |  |  |  |
| Q1 | Reference | Reference |  |  |  |  | Reference | Reference |  |  |  |  | Reference | Reference | |  |  |  |  |
| Q2 | 0.92 | 0.75, 1.14 | 0.5 |  |  |  | 0.91 | 0.74, 1.12 | 0.4 |  |  |  | 1.02 | 0.84, 1.24 | | 0.8 |  |  |  |
| Q3 | 0.81 | 0.69, 0.95 | 0.011 |  |  |  | 0.80 | 0.68, 0.94 | 0.007 |  |  |  | 0.93 | 0.79, 1.08 | | 0.3 |  |  |  |
| Q4 | 0.55 | 0.45, 0.68 | <0.001 |  |  |  | 0.55 | 0.45, 0.68 | <0.001 |  |  |  | 0.69 | 0.56, 0.85 | | <0.001 |  |  |  |
|  |  |  |  |  |  |  |  |  |  |  |  |  |  |  | |  |  |  |  |

Model 1 adjusted for age. Model 2 adjusted for sex, age, and race/ethnicity. Model 3 adjusted for sex, age, race/ethnicity, educational level, marital status, poverty-to-income ratio, smoking history, alcohol consumption, health insurance status, healthcare utilization, and history of diabetes.

Abbreviation: HR, hazard ratio.

**Supplementary Table 10: Hazard Ratios for Mortality by Joint Categories of Nutrition-OBS and Lifestyle-OBS (Dichotomized at Median)**

|  | **P for interaction = 0.988** | | | **P for interaction = 0.584** | | |
| --- | --- | --- | --- | --- | --- | --- |
|  | **All-Cause Mortality** | | | **Cardiovascular Mortality** | | |
| **Characteristic** | **N** | **HR (95% CI)** | **p-value** | **N** | **HR (95% CI)** | **p-value** |
| Low Nutrition / Low Lifestyle | 3,499 | 1.00 (Reference) | — | 3,023 | 1.00 (Reference) | — |
| High Nutrition / Low Lifestyle | 1,324 | 0.80 ( 0.69, 0.94) | 0.006 | 1,098 | 0.78 ( 0.56, 1.09) | 0.140 |
| Low Nutrition / High Lifestyle | 3,020 | 0.99 ( 0.84, 1.17) | 0.896 | 2,686 | 0.88 ( 0.64, 1.22) | 0.454 |
| High Nutrition / High Lifestyle | 1,462 | 1.00 ( 0.75, 1.33) | 0.988 | 1,293 | 0.86 ( 0.49, 1.49) | 0.584 |

All models are adjusted for age, sex, race/ethnicity, marital status, education, poverty-income ratio, drinking, insurance, healthcare, smoking, and diabetes. OBS subscores were dichotomized at their respective medians.

**Supplementary Table 11 Mediation analysis of oxidative balance score (OBS) and mortality risk among U.S. adults with hypertension (N = 9,305): Exploring the roles of low-grade systemic inflammation and multi-organ function.**

|  |  |  | Estimated proportion |  |
| --- | --- | --- | --- | --- |
| Variable | Total Effect | Direct Effect | Indirect Effect | % Mediated |
| eGFR | -0.00320 | -0.00314 | -0.00006 | 2.08% |
|  | (-0.0001,0.00) | (-0.00409,0.00) | (-0.00010,0.00) |  |
| P value | <0.001 | <0.001 | <0.001 | <0.001 |
| UACR | -3.20e-03 | -3.14e-03 | -5.39e-05 | 1.55% |
|  | (-4.22e-03,0.00) | (-4.19e-03,0.00) | (-1.21e-04,0.00) |  |
| P value | <0.001 | <0.001 | <0.001 | <0.001 |
| FIB-4 | -3.08e-03 | -3.12e-03 | 3.75e-05 | -1.33% |
|  | (-4.07e-03,0.00) | (-4.11e-03,0.00) | (-2.83e-03,0.00) |  |
| P value | 0.44 | <0.001 | <0.001 | 0.44 |
| NLR | -3.12e-03 | -3.05e-03 | -7.31e-05 | 2.02% |
|  | (-3.93e-03,0.00) | (-3.89e-03,0.00) | (-1.59e-04,0.00) |  |
| P value | <0.001 | <0.001 | 0.04 | 0.04 |
| SIRI | -0.003118 | -0.003006 | -0.000112 | 3.17% |
|  | (-0.003963,0.00) | (-0.003864,0.00) | (-0.000210,0.00) | - |
| P value | <0.001 | <0.001 | <0.001 | <0.001 |
| SUA | -0.003164 | -0.002996 | -0.000168 | 5.47% |
|  | (-0.004249,0.00) | (-0.004106,0.00) | (-0.000283,0.00) |  |
| P value | <0.001 | <0.001 | 0.04 | 0.04 |
|  |  |  |  |  |

Abbreviation: CI, confidence interval.

Adjusted for sex, age, race/ethnicity, educational level, marital status, poverty-to-income ratio, smoking history, alcohol consumption, health insurance status, healthcare utilization, and history of diabetes

**Supplementary Figure 1** **Mediation analysis of oxidative balance score (OBS) and mortality risk in U.S. adults with hypertension (N = 9,305): Exploring the role of low-grade systemic inflammation and multi-organ function in this relationship.**

**
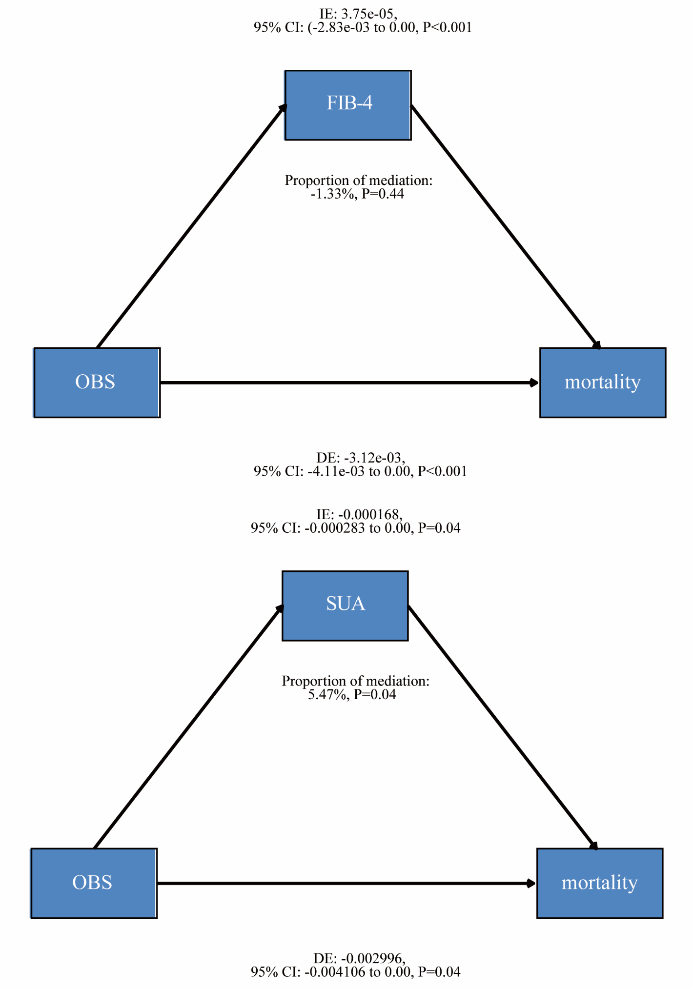

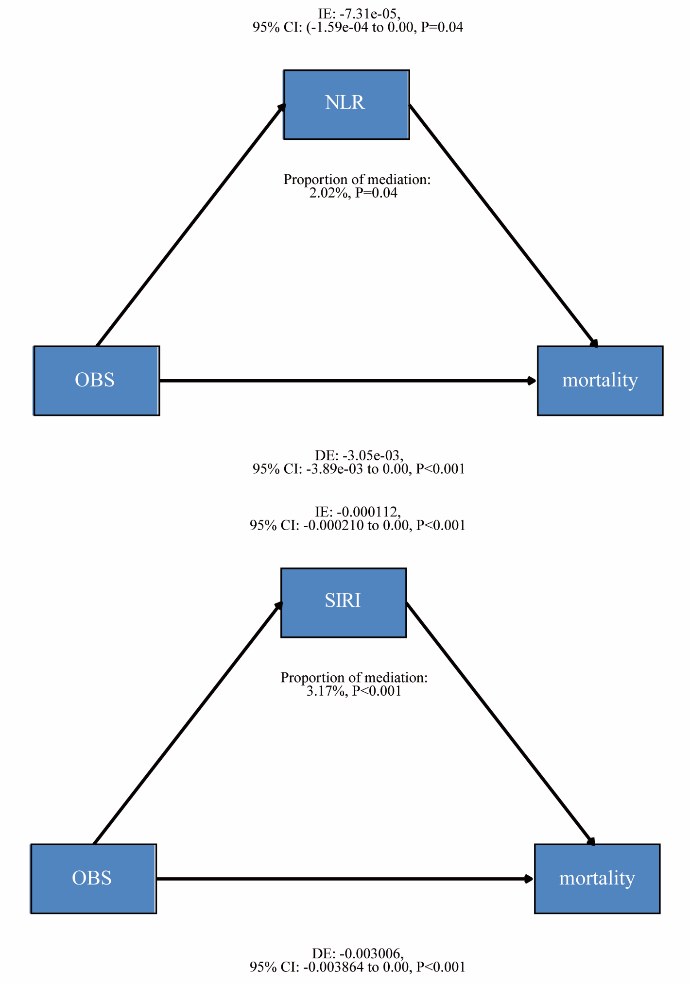

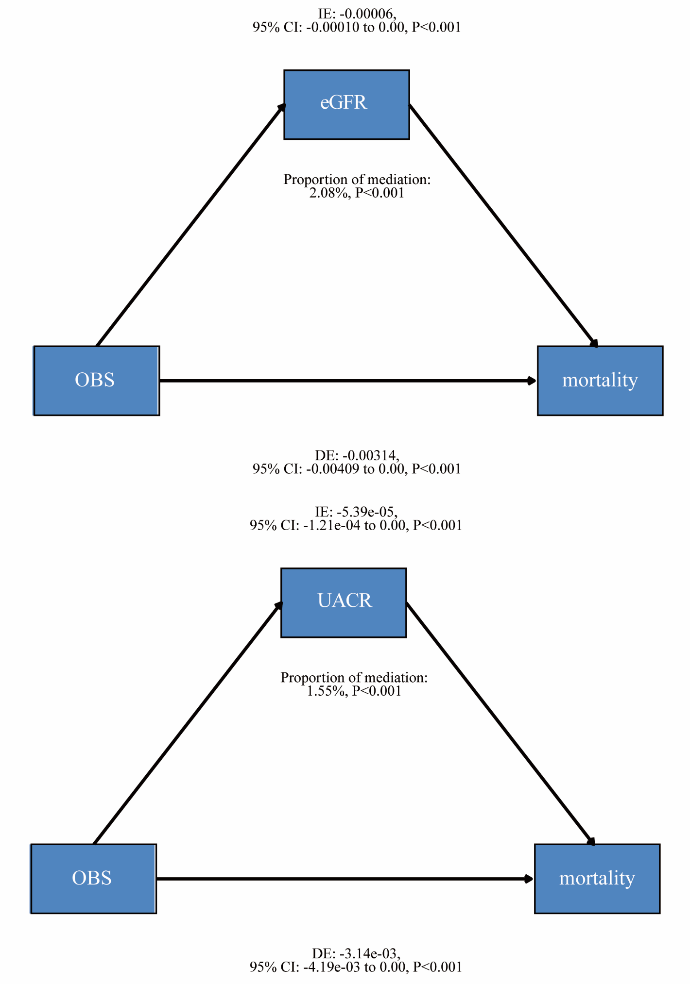
**

Abbreviation: CI, confidence interval.

Adjusted for sex, age, race/ethnicity, educational level, marital status, poverty-to-income ratio, smoking history, alcohol consumption, health insurance status, healthcare utilization, and history of diabetes.

**Supplementary** **Information Variable calculation formula.**

The eGFR formula used in this study was: eGFR = 175 × Serum creatinine−1.154 × Age−0.203 × 0.742(if female) × 1.212(if Black)

The FIB-4 index was calculated based on the standardized equation:

$$\text{ FIB-4 = }\frac{\text{Age (years) × AST (U/L) }}{\text{ PLA × }\sqrt{\text{ALT (U/L)}}}$$

Where AST denotes aspartate aminotransferase, ALT refers to alanine aminotransferase, and PLA signifies platelet count.
